# Florfenicol-induced dysbiosis impairs intestinal homeostasis and host immune system in laying hens

**DOI:** 10.1186/s40104-025-01186-w

**Published:** 2025-04-14

**Authors:** Keesun Yu, Inhwan Choi, Minseong Kim, Young Jin Pyung, Jin-Sun Lee, Youbin Choi, Sohyoung Won, Younghoon Kim, Byung-Chul Park, Seung Hyun Han, Tae Sub Park, Tina Sørensen Dalgaard, Cheol-Heui Yun

**Affiliations:** 1 Department of Agricultural Biotechnology, and Research Institute of Agriculture and Life Sciences, Seoul National University, Seoul, 08826 Republic of Korea; 2 Department of Oral Microbiology and Immunology, and Dental Research Institute, School of Dentistry, Seoul National University, Seoul, 08826 Republic of Korea; 3 Interdisciplinary Program in Bioinformatics, Seoul National University, Seoul, Republic of Korea; 4 Graduate School of International Agricultural Technology and Institute of Green-Bio Science and Technology, Seoul National University, Pyeongchang-Gun, Gangwon-Do, 25354 Republic of Korea; 5 Department of Animal and Veterinary Sciences, Aarhus University, Tjele, Denmark

**Keywords:** Antibiotics-induced dysbiosis, Avian immunology, Avian pathogenic *Escherichia coli*, Gut homeostasis, Laying hen, Short chain fatty acids

## Abstract

**Background:**

Despite growing concerns about the adverse effects of antibiotics in farm animals, there has been little investigation of the effects of florfenicol in laying hens. This study examined the effect of florfenicol on the intestinal homeostasis, immune system, and pathogen susceptibility of laying hens.

**Results:**

The oral administration of florfenicol at field-relevant levels for 5 d resulted in a decrease in the gut microbiota genera *Lactobacillus, Bacillus,* and *Bacteroides*, indicating the development of intestinal dysbiosis. The dysbiosis led to decreased mRNA levels of key regulators peroxisome proliferator-activated receptor gamma (*PPAR-γ*) and hypoxia-inducible factor-1α (*HIF-1α*), compromising intestinal hypoxia. Intestinal homeostasis was also disrupted, with decreased expression of Occludin and Mucin 2 *(Muc2)* genes combined with increased gut epithelial permeability. The breakdown in intestinal homeostasis and immune function provided a favorable environment for opportunistic bacteria like avian pathogenic *Escherichia coli* (APEC), culminating in systemic infection. Immunologically, florfenicol treatment resulted in increased proportion and absolute number of MRC1L-B^+^ monocytes/macrophages in the spleen, indicating an exacerbated infection. Furthermore, both the proportion and absolute number of γδ T cells in the lamina propria of the cecum decreased. Treatment with florfenicol reduced butyrate levels in the cecum. However, the administration of butyrate before and during florfenicol treatment restored factors associated with intestinal homeostasis, including *PPAR-γ, *Occludin, and *Muc2*, while partially restoring *HIF-1α*, normalized intestinal hypoxia and gut permeability, and reversed immune cell changes, suppressing APEC systemic infection.

**Conclusion:**

The uncontrolled and widespread use of florfenicol can negatively affect intestinal health in chickens. Specifically, florfenicol was found to impair intestinal homeostasis and immune function in laying hens, including by reducing butyrate levels, thereby increasing their susceptibility to systemic APEC infection. The development of strategies for mitigating the adverse effects of florfenicol on gut health and pathogen susceptibility in laying hens is therefore essential.

**Supplementary Information:**

The online version contains supplementary material available at 10.1186/s40104-025-01186-w.

## Background

Avian pathogenic *Escherichia coli* (APEC), classified as extraintestinal pathogenic *E*. *coli* (ExPEC), comprise a group of opportunistic pathogens that cause inflammation, diarrhea, and intestinal hemorrhage in birds [1]. While they are typically found as commensal bacteria in the gut, under conditions such as reduced microbial competition or increased nutritional availability, they can cause widespread disease in poultry flocks, resulting in detrimental economic crisis in the poultry industry worldwide [2]. APEC can become airborne and survive in dry environments such that, thus far, systemic infections in poultry (colibacillosis) have mostly been attributed to respiratory infection [3]. However, while mouse studies have shown that *E*. *coli* can translocate from the intestine into the bloodstream, potentially causing systemic diseases [4], the intestinal transmission of colibacillosis in chickens has not been explored. Additionally, the most prevalent APEC serotypes threatening the poultry industry are O1, O2, and O78, which have comparable phylogenetic backgrounds and share several drug-resistance genes and virulence genes with human ExPEC [5]. The similarities underscore the potential for zoonotic transmission of APEC and thus the importance of controlling infections to protect the poultry industry and human health.

Under physiological conditions, the lumen of the gastrointestinal tract maintains a highly hypoxic environment, which is essential for sustaining a healthy gut microbiota [6]. Homeostasis in the gut is maintained through interactions between the microbiota and the host, with significant research in mice focusing on how microbiota-derived metabolites contribute to this balance [7, 8]. For instance, short-chain fatty acids (SCFAs), particularly butyrate, regulate the metabolism and oxygen consumption of intestinal epithelial cells, thereby sustaining the hypoxic environment in the gut and supporting barrier function [9]. However, research on intestinal homeostasis in poultry, including the metabolism of intestinal epithelial cells, oxygen consumption, and the maintenance of hypoxic conditions, remains limited. Moreover, gut microbiota-host interactions extend beyond gut homeostasis as they also influence the systemic immune system [10, 11], but studies exploring the effects of gut microbiota-host interactions on systemic immune responses are also lacking. Understanding the factors that regulate intestinal homeostasis and systemic immunity in chickens is crucial for improving the growth and immune function of these important farm animals, as it will also increase their health and productivity.

While antibiotics are essential for the treatment of many bacterial infections, in both animals and humans, there is ample evidence of their adverse effects. Antibiotic use can cause dysbiosis, defined as an imbalance or disruption in the normal gut microbiota, which frequently causes digestive or health problems. Antibiotic-induced dysbiosis can promote pathobiont growth [12, 13], deplete beneficial microbes [14], and reduce microbial diversity [15], all of which have negative physiological impacts on the host. Other intestinal effects of antibiotics include the reduced expression of tight junction proteins [16, 17], disruption of the mucus barrier [18], impairment of goblet cell function [19], and a weakening of the gut barrier function in the host [20]. Additionally, antibiotic-induced dysbiosis is linked to altered gastrointestinal immunity, including an increase in inflammatory responses. In the dysbiotic state caused by antibiotics, intestinal Th1 cells expand [21] concomitantly with a reduction in Th17 [22] and Treg cells [23], and increased production of pro-inflammatory cytokines [24], leading to an acute inflammatory response in the intestine. This negative impact of antibiotic-induced dysbiosis on the host gastrointestinal immune system may persist long after antibiotic administration is discontinued [25].

Florfenicol, a broad-spectrum antibiotic effective against both Gram-negative and Gram-positive bacteria, is the primary choice for managing colibacillosis in broiler and pre-laying pullets [26, 27]. In the poultry industry, it is used not only in disease treatment but also prophylactically; however, this indiscriminate use may have adverse consequences, including the selection of antibiotic-resistant bacteria [28] and the development of dysbiosis [29]. Studies in poultry have demonstrated the adverse effects of florfenicol administration, including gut microbial dysbiosis and associated metabolomic changes [30], a disruption of immune function [31], altered drug metabolism and lipid synthesis in the liver [32], decreased growth [33], and early embryonic death [34]. Nonetheless, the adverse effects induced by antibiotic-driven dysbiosis have mostly been studied in rodents, research on antibiotic-induced dysbiosis and its effects on intestinal homeostasis and metabolites in chickens remain very limited. Furthermore, these disruptions may cause malfunctions in intestinal epithelial barriers, which serve as the body’s first line of defense and may have a substantial impact on pathogen prevention. However, there has been a significant lack of research into how these changes affect the systemic immune system during such infections.

Therefore, the present study investigated the impact of florfenicol-induced dysbiosis on intestinal homeostasis, including intestinal hypoxic conditions and gut integrity, in laying hens. Since florfenicol has also been shown to disrupt the intestinal epithelial barrier and induce systemic APEC infection, thus compromising the host’s first line of defense, its effects on host immune responses and systemic immunity were examined as well. The factors involved in the identified antibiotic-sensitive processes were elucidated by analyzing changes in intestinal metabolites, with a particular focus on butyrate levels. Butyrate administration was found to alleviate florfenicol-induced changes in intestinal homeostasis and the host immune system in addition to restoring pathogen resistance.

## Methods

### Animal experiment

Fertile eggs from White Leghorn chickens, obtained at the animal farm of Seoul National University, Pyeongchang, Korea, were incubated at 37 °C for 3 weeks. The newly hatched chicks were housed in cages and provided with feed and water ad libitum for 2 weeks without any additives or vaccination. The experiment was approved by the Institutional Animal Care and Use Committee of Seoul National University (IACUC No. SNU-230818-1).

To investigate the effect of florfenicol on intestinal homeostasis, 2-week-old chickens (*n* = 6 per group) were treated with florfenicol (Sigma-Aldrich, Saint Louis, MO, USA) at a dose of 30 mg/kg body weight via oral gavage for 5 d. During the withdrawal phase, florfenicol-treated chickens were housed for 2 d with no further florfenicol treatment, until florfenicol was no longer detectable in their intestines [35]. To assess the role of butyrate in restoring intestinal homeostasis impaired by florfenicol treatment, butyrate (100 mmol/L, Sigma-Aldrich) was administered to florfenicol-treated chickens via drinking water from the beginning to the completion of the experiment (FFC + B group).

To investigate the effect of florfenicol on APEC infection, chickens (*n* = 6 per group) were pre-infected with 1 × 10^11^ colony-forming units (CFU) of APEC (Korean Collection for Type Cultures, KCTC 2441, O1:K1) in 500 μL of saline via oral gavage to ensure colonization in all chickens, replicating a typical situation in which florfenicol is administered to flocks after APEC infection, by which time most chickens have already been colonized through fecal transmission. The bacteria were grown aerobically at 37 °C in nutrient broth and nutrient agar plates (BD Biosciences, Franklin Lakes, NJ, USA). Based on the CFU counts in the cecum and feces, 3 d were determined to be sufficient for APEC colonization in the cecum (data not shown). Thus, 3 d after the initial APEC infection, the chickens were treated (T.F.T group) or not (T.T group) with 30 mg florfenicol/kg body weight, administered daily for 5 d via oral gavage. During the withdrawal phase, florfenicol-treated chickens were housed for 2 d without additional florfenicol treatment. Following the withdrawal phase, the chickens were re-infected with APEC (1 × 10^11^ CFU/mL) via oral gavage to simulate field conditions of ongoing APEC exposure after florfenicol treatment. The chickens were euthanized 3 d later. To assess the role of butyrate in modulating susceptibility to APEC infection, florfenicol-treated chickens were administered butyrate (100 mmol/L) via their drinking water throughout the experiment (T.F.T + B group).

### Isolation and counting of bacteria

After the final APEC infection, the cecal contents of the T.T and T.F.T groups, as well as the cecal contents of the FFC and FFC + B groups after a 2-d withdrawal phase, were aseptically isolated, weighed, and suspended in 0.1% peptone water (BD Biosciences). The suspension was then passed through a 100-µm cell strainer (BD Biosciences) and serially diluted tenfold in 0.1% peptone water. The diluted samples were plated on MRS agar and MacConkey agar (both from BD Biosciences) and incubated at 37 °C overnight, after which the number of colony-forming units was counted.

### Bacteria translocation

The ability of APEC to induce systemic infection was determined by examining the presence in the spleen. A spleen suspension in phosphate-buffered saline (PBS) was prepared from aseptically isolated chicken spleens and then passed through a 40-µm cell strainer (BD Biosciences). Overnight cultures of the cells on MacConkey agar (BD Biosciences) were incubated at 37 °C and then analyzed for infection by determining the number of CFU.

### Quantitative real-time PCR

The cecum was washed with PBS and cut into smaller pieces (0.2–0.5 cm). Total RNA was isolated using TRIzol® reagent (Invitrogen, Carlsbad, CA, USA) according to the manufacturer’s instructions. For bacterial samples, 200 µL of preheated (95 °C) TRIzol® Max from a bacterial RNA isolation kit (Ambion, Austin, TX, USA) was added to the bacterial colonies isolated from the spleen suspensions and incubated at 95 °C for 4 min, followed by the addition of 1 mL of TRIzol® reagent [36]. Extracted RNA was reverse-transcribed into cDNA, and PCR was performed using the StepOne Plus real-time PCR system (Applied Biosystems, Foster City, CA, USA). The PCR was carried out in a 96-well reaction plate (Applied Biosystems) with 9 µL of SYBR® Green PCR master mix (Applied Biosystems), 1 µL of cDNA template, 8 µL of nuclease-free distilled water (Sigma-Aldrich), and 1 µL of the respective forward or reverse primers for each gene (Table 1). Relative expression levels of the target genes were calculated using the 2^-ΔΔ*CT*^ method. The expression of all target genes was normalized to β-actin and 16S rRNA levels.

**Table 1:** Nucleotide sequence of the real-time PCR primers

**Gene**	**Nucleotide sequence (5´→3´)**	**Gene ID**
*PPAR-γ*	F: TACATAAAGTCCTTCCCTCTGA	373928
	R: TCCAGTGCATTGAACTTCACAG	
*HIF-1α*	F: ATCAGAGTGGTTGTCCAGCAG	374177
	R: CAGTCCAAGCCCACCTTACT	
Occludin	F: CGAGTTGGATGAGTCCCAGT	396026
	R: TTGATGCTGTCCATCTCAGC	
*Muc2*	F: GATCTTCCTTGACAGCTTTTGAACT	423101
	R: AAATGATCCATAGGTGTATGCAACTC	
β-Actin	F: CAACACAGTGCTGTCTGGTG	396526
	R: ATCGTACTCCTGCTTGCTGA	
*wzx*	F: GTGAGCAAAAGTGAAATAAGGAACG	75172157
	R: CGCTGATACGAATACCATCCTAC	
*neuC1*	F: AGGTGAAAAGCCTGGTAGTGTG	69473208
	R: GGTGGTACATCCCGGGATGTC	
16S rRNA	F: AGAGTTTGATCMTGGCTCAG	2827929
	R: CTGCTGCSYCCCGTAG	

### Gut permeability test

The chickens were fasted for 24 h and then administered FITC-dextran (4 kDa, Sigma-Aldrich) dissolved in PBS via oral gavage at a dose of 8.5 mg/kg body weight. Blood samples were obtained from the heart 7 h post-administration and centrifuged to obtain serum. Serum FITC levels were measured using a multi-plate reader (Molecular Devices, San Jose, CA, USA) at excitation/emission wavelengths of 485/528 nm and calculated using a standard curve.

### Immune cell isolation

The cecum was removed, washed with PBS, cut into small pieces (0.5–1 cm), and incubated in 10 mL of Mg^2+^- and Ca^2+^-free HBSS (Gibco, Grand Island, NY, USA) containing 10% fetal bovine serum (FBS, Gibco), 10 mmol/L HEPES (Invitrogen, Carlsbad, CA, USA), and 2 mmol/L EDTA (Invitrogen) for 1 h at 37 °C using a magnetic stirrer to isolate the intraepithelial lymphocytes. The cells were passed through a 100-µm strainer and washed with PBS. The remaining tissue from the cecum was incubated in 10 mL of Mg^2+^- and Ca^2+^-free HBSS containing 10% FBS, 10 mmol/L HEPES, 0.5 mg/mL collagenase type IV (Sigma-Aldrich) and 50 µg/mL DNase I (Sigma-Aldrich) for 1 h at 37 °C with a magnetic stirrer to isolate lamina propria lymphocytes. The suspension was passed through a 100-µm cell strainer and the cells were isolated by density gradient centrifugation for 25 min at 400 × *g* and 18 °C using 40% and 70% Percoll (Cytiva, Marlborough, MA, USA).

The spleen was removed from the chicken, placed in RPMI 1640 (Gibco) containing 10% FBS, passed through a 40-µm strainer, and then centrifuged. The isolated cells were incubated for 10 min at 4 °C with 5 mL of ACK lysis buffer containing 0.15 mol/L ammonium chloride (Sigma-Aldrich), 0.01 mol/L potassium bicarbonate (Sigma-Aldrich), and 0.0001 mol/L EDTA, with the pH adjusted to 7.2–7.4. Then, 5 mL of PBS was added to the cell suspension, which was then centrifuged to collect the cell pellet.

### Flow cytometry analysis

Isolated LPLs and splenocytes were plated in a 96-well V-bottom plate (Thermo Fisher Scientific, Waltham, MA, USA) at a density of 1 × 10^6^ cells per well. The cells were processed as follows: T cells were stained with the LIVE/DEAD™ Fixable Near-IR dead cell stain kit (Thermo Fisher Scientific) and mouse anti-chicken CD3-PACBLU (CT-3), CD4-PE/CY7 (CT-4), CD8a-PE (CT-8), CD8b-biotinylated (EP-42), CD45-SPRD (LT-40), and TCRγδ-FITC (TCR1) (all from Southern Biotechnology, Birmingham, AL, USA) for 20 min at 4 °C in the dark. The cells were then washed with PBS containing 5% FBS, stained with Brilliant Violet 605™ streptavidin (BioLegend, San Diego, CA, USA) for 20 min at 4 °C in the dark, and washed with PBS containing 5% FBS. Monocytes/macrophages were stained with the LIVE/DEAD™ Fixable Near-IR dead cell stain kit, CD45-SPRD (LT-40), MHC class II-FITC (2G11), and MRC1L-B-PE (KUL01) (all from Southern Biotechnology) for 20 min at 4 °C in the dark and then washed with PBS containing 5% FBS [37]. The cells were acquired using a FACS Canto II (BD Biosciences) and analyzed using FlowJo software (Tree Star Inc., Ashland, OR, USA).

### Hypoxia staining

For imaging-based detection of hypoxia, the chickens were euthanized 1 h after an intraperitoneal injection of 60 mg/kg of pimonidazole HCl (Hypoxyprobe, Burlington, MA, USA). Cecum samples were washed with PBS, fixed in 4% buffered paraformaldehyde (Sigma-Aldrich), dehydrated sequentially in 20% and 30% sucrose (Sigma-Aldrich) overnight, and then embedded in a frozen section compound (Leica Biosystems, Nussloch, Germany). Transverse slices of 7 µm thickness were cut, thawed, and incubated with rat anti-pimonidazole monoclonal antibody MAb1 (Hypoxyprobe), followed by a secondary FITC-conjugated goat anti-rat IgG antibody (Jackson ImmunoResearch, West Grove, PA, USA) and Hoechst counter-staining. The images were captured using a digital upright fluorescence microscope (Olympus Corporation, Hachioji, Tokyo, Japan).

### Measurement of SCFAs

Cecum contents were diluted tenfold in deuterium oxide (Sigma-Aldrich) and filtered through a 0.45-µm filter (Sartorius, Otto-Brenner-Str, Göttingen, Germany). Metabolite changes were analyzed using 600 MHz ^1^H-nuclear magnetic resonance (NMR) spectroscopy (Bruker, Billerica, MA, USA). SCFA concentrations were determined by diluting the cecum samples tenfold in distilled water, followed by filtration through a 0.45-µm filter, and high performance liquid chromatography (HPLC) (Thermo Fisher Scientific) analysis.

### Shotgun metagenome sequencing

Genomic DNA was extracted from the cecal contents using the QIAamp Fast DNA stool mini kit (Qiagen, Hilden, Germany). Sequencing libraries were prepared using the TruSeq Nano DNA library prep kit (Illumina, CA, USA). The adaptor-ligated DNA was PCR-amplified over eight cycles using a high-fidelity polymerase. Library fragment size and quantity were determined using Tapestation4200 (Agilent Technologies, CA, USA) and the KAPA library quantification kit (KAPA Biosystems), respectively. Libraries with an insert size of ~ 650 bp were constructed for each group and sequenced using the Nextseq2000 platform (Illumina, CA, USA). Raw reads were processed for quality control and adapter removal using Trimmomatic v0.39 (AM Bolger, M Lohse, B Usadel, 2014). To avoid potential PhiX contamination, trimmed reads were aligned with the PhiX reference genome (NC_001422.1) using BWA v0.7.17-r1188 and the aligned reads were filtered using SAMtools v1.15.1. Taxonomic classification was performed using Kraken2 v2.1.2 and species abundance was estimated using Bracken v2.55. The rtk v0.2.6.1 R package was used to measure α-diversity based on the Shannon index. The phyloseq package v1.34.0 in R was used to determine β-diversity by calculating weighted and unweighted UniFrac distances.

### Statistical analyses

The number of experimental units was pre-determined through a power analysis to ensure sufficient statistical significance. Data were examined using a Completely Randomized Design (CRD) [38–40], wherein the chickens were randomly assigned to the different groups to minimize bias and ensure reliable comparisons. Results are presented as the mean ± SEM. Statistical analysis was performed using GraphPad Prism (GraphPad Software, San Diego, USA). Student’s *t*-test was used to compare two groups, and a one-way ANOVA followed by Tukey’s multiple-comparison test, using Prism 10, to determine differences among multiple groups. Differences were considered significant at *P < 0.05, **P < 0.01, or ***P < 0.001.

For the analysis of correlations between microbiota and metabolites, we first determined the proportional abundance of each taxon and excluded any phylum, genus, or species with a minimum abundance below 0.001 or detected in fewer than three samples (prevalence < 0.375). This filtering yielded 338 taxa, for which we calculated Spearman correlations (involving three metabolites) and corresponding *P*-values using the cor.test function in R. Correlations with *P*-values below 0.01 were then visualized in a heatmap generated by the ComplexHeatmap package. For the correspondence analysis (CCA) analysis, we focused on 165 species meeting the same abundance and prevalence criteria. The analysis was performed at the species level using the vegan package in R, and plots were created with ggplot2. Significant results from the Spearman correlation analysis (*P* < 0.01) were labeled in the CCA plot. Significance levels are indicated as follows: *P < 0.05, **P < 0.01, ***P < 0.001.

## Results

### Administration of florfenicol alters the gut microbiota composition

**Fig. 1:**
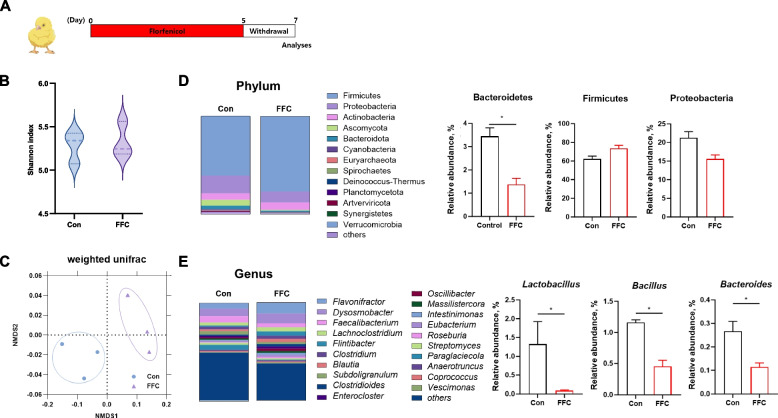
Composition of microbial communities in the ceca. **A** Schematic representation of the study design. Florfenicol was administered for 5 d followed by a 2-d withdrawal period. **B** Violin plots of Shannon index in abundances in the control (*n* = 3) and florfenicol-treated (*n* = 3) groups. **C** A principal coordinate analysis with weighted Unifrac distances was used to assess β-diversity, based on the relative species abundances in the control (*n* = 3) and florfenicol-treated (*n* = 3) groups. **D** and **E** The composition of the gut microbiota at the phylum and genus levels in chickens treated with florfenicol (FFC) or PBS. Statistical significance was determined in a *t*-test; *P < 0.05

In most studies of antibiotics-induced dysbiosis in chickens, antibiotics are administered for more than a week [30]. However, to closely mimic field conditions, the protocol used in our study consisted of 5 d antibiotic administration followed by a 2-d withdrawal phase (Fig. 1A). The induction of dysbiosis was confirmed by analyzing the gut microbiota in the cecum after the withdrawal phase. Consistent with previous reports of florfenicol-induced dysbiosis in chickens, the α-diversity of gut microbiota communities, measured based on the Shannon index, was unaffected by florfenicol treatment (Fig. 1B). A principal coordinate analysis (PCoA) was performed using the weighted UniFrac method to evaluate the similarity of bacterial communities between groups (Fig. 1C). The results suggested that the antibiotic group is significantly separated from the control group. An analysis of the bacterial composition revealed differences in the microbial communities between the control and florfenicol-treated groups. At the phylum level, 38 phyla, including Firmicutes, Proteobacteria, Bacteroidetes, Actinobacteria, Acidobacteria, Artverviricota, Ascomycota, Cyanobacteria, Fusobacteria, and Euryarchaeota, were identified. Firmicutes, Proteobacteria, and Bacteroidetes predominated, accounting for > 80% of the total microbial community. Florfenicol treatment led to a decrease in Proteobacteria and an increase in Firmicutes and statistically significant trends of a decrease in Bacteroidetes (Fig. 1D). At the genus level, 981 genera were identified, with *Paraglaciecola*, *Faecalibacterium*, *Dysosmobacter*, and *Flavonifractor* as the most abundant. Florfenicol treatment decreased the abundance of *Lactobacillus*, *Bacillus*, and *Bacteroides* (Fig. 1E). These findings indicated that 5 d of florfenicol treatment followed by a 2-d withdrawal phase induces dysbiosis in the chickens.

### Florfenicol-induced dysbiosis impairs gut homeostasis

During homeostasis, the gut microbiota produces SCFAs, which contribute to maintenance of a hypoxic state within the lumen of the intestine [41]. In turn, hypoxia induces the expression of hypoxia-inducible factor-1 (HIF-1) and thus the expression of several hypoxia-related genes in intestinal epithelial cells [42, 43]. SCFAs also activate peroxisome proliferator-activated receptor gamma (PPAR-γ), which enhances mitochondrial β-oxidation and increases oxygen consumption, thus reinforcing the hypoxic state of the intestinal lumen [9].

**Fig. 2:**
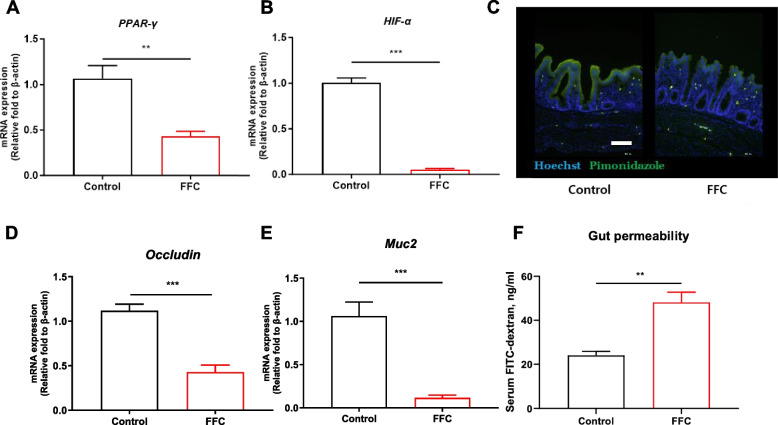
Reduced hypoxic conditions and increased intestinal permeability in florfenicol-treated chickens. **A** and **B** Expression levels of *PPAR-γ* and *HIF-1α* in chicken cecum (*n* = 6 chickens per group) treated with PBS or florfenicol. **C** The maintenance of cecal hypoxia was examined by detecting the binding of pimonidazole (scale bar = 100 µm). **D** and **E** Cecal Occludin and *Muc2* gene expression were measured in chickens fasted for 24 h before oral FITC-dextran administration. **F** The amount of FITC in the serum was measured 7 h later to assess gut permeability. FFC, florfenicol. Results are presented as the mean ± SEM. Statistical differences were determined in a *t*-test; *P < 0.05, **P < 0.01, ***P < 0.001

To determine whether florfenicol treatment disrupts intestinal homeostasis, changes in the expression of *HIF-1α* and *PPAR-γ* were examined. The results showed notable reductions in *HIF-1α* and *PPAR-γ* expression in the florfenicol-treated group compared to the control group (Fig. 2A and B). Oxygen accumulation in the cells was detected using pimonidazole, which forms covalent bonds with macromolecules in hypoxic cells [44]. Consistent with the changes in *HIF-1α* and *PPAR-γ* expression, pimonidazole levels were lower in the florfenicol-treated group than in the control group (Fig. 2C). These findings demonstrated that intestinal hypoxia is impaired in the ceca of chickens treated with florfenicol.

HIF-1α and PPAR-γ maintain gut integrity by regulating the function of tight junctions [45, 46] and the synthesis of mucin [9] in intestinal epithelial cells. In the florfenicol-treated group, however, the expression of Occludin and Mucine2 (*Muc2)*, genetic markers of these functions, was reduced (Fig. 2D and E).

Following its oral administration, 4-kDa FITC-dextran passively crosses the intestinal epithelium and can be detected in the serum, enabling an assessment of gut permeability [47]. The increase in serum dextran levels in the florfenicol-treated chickens indicated an increase in intestinal permeability (Fig. 2F). These findings demonstrated that florfenicol treatment causes an imbalance in the gut microbiota of chickens, resulting in a state of dysbiosis that disrupts cecal homeostasis by impairing epithelial hypoxia and damaging intestinal integrity.

### Florfenicol-induced dysbiosis increases the susceptibility of chickens to systemic APEC infection

Impaired hypoxic conditions in the gut create an environment conducive to *E*. *coli* proliferation, while a reduced gut integrity weakens host defenses against pathogen invasion. We therefore examined whether florfenicol-induces dysbiosis facilitates systemic APEC infection.

**Fig. 3:**
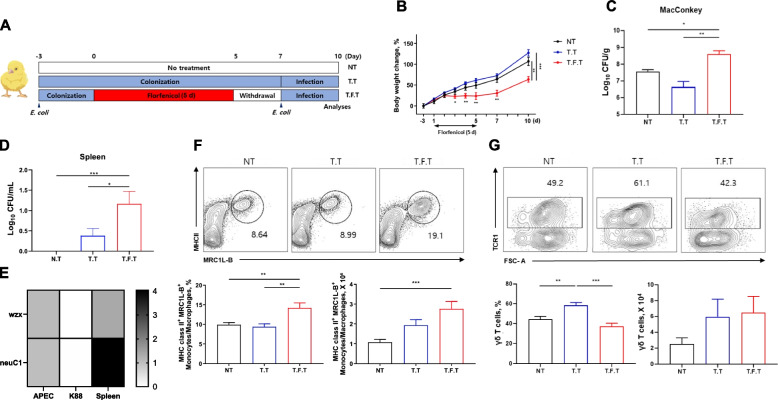
Chickens with dysbiosis are more susceptible to systemic avian pathogenic *Escherichia coli* (APEC) infection and changes in immune cell composition. **A** Chickens (*n* = 6 per group) were infected with APEC 3 d prior to florfenicol treatment and then re-infected following the withdrawal phase. **B** and **C** Body weight changes (**B**) and pathobionts in the cecal contents (**C**) were examined 3 dpi by plating the cecal contents on MacConkey agar plates. **D** Systemic APEC infection was quantified by determining the mean log_10_ CFU/mL in a splenic suspension plated on MacConkey agar plates. **E** The serotype of the APEC colonies was identified by comparing antigen transcripts (*wzx* and *neuC1*). APEC and *E*. *coli* K88 from stock cultures served as positive and negative controls, respectively. **F**–**G** Changes in the percentage and absolute numbers of splenic monocytes/macrophages and lamina propria γδ T cells were determined. The frequency of monocytes/macrophages was expressed as a percentage of the total CD45^+^ population, whereas the frequency of γδ T cells was expressed as a percentage of the CD45^+^CD3^+^ population. NT, non-treated. T.T, APEC double infection without florfenicol treatment. T.F.T, APEC double infection with florfenicol treatment. Significance differences were examined by using Tukey test, with significance levels denoted as follow: *P < 0.05, **P < 0.01, ***P < 0.001

To reproduce the opportunistic nature of APEC, we developed a model to ensure colonization prior to florfenicol treatment. Thus, an initial infection was established before florfenicol administration in both the treated (T.F.T) and untreated (T.T) groups, followed by a secondary infection after florfenicol treatment (Fig. 3A). This protocol resulted in a lower body weight gain from 3 to 10 d (Fig. 3B) and a higher CFU count in the cecal contents of the T.F.T group (Fig. 3C) than in the other groups.

Systemic APEC infection was confirmed by examining bacterial translocation in the spleen. Despite pre-colonization with APEC, the bacteria were not detected in the spleen during the early stages of bacterial infection (1 d post-infection, 1 dpi) in any of the groups (Fig. S1A). However, at 3 dpi, bacteria were observed in the spleens of the T.T group and especially in the spleens of the T.F.T group (Fig. 3D). This confirms that the treatment worsened the infection, as opposed to the control group that was not infected with bacteria. Analysis of the mRNA levels of *wzx*, encoding the O1 antigen of lipopolysaccharide, and of *neuC1*, encoding the K1 antigen of the capsule, in bacterial RNA from APEC O1:K1, *E*. *coli* K88, *E*. *coli* K99, and *Salmonella* Enteritidis revealed that these genes were expressed exclusively in APEC O1:K1 (Fig. S1B and C). Colonies isolated from the spleen expressed both *wzx* and *neuC1* (Fig. 3E), thus demonstrating that the bacteria responsible for the systemic infection were APEC serotype O1:K1, the strain used in this study to infect the chickens.

The effects of APEC infection on local and systemic immune responses were examined by isolating immune cells from the spleen and cecum (Figs. S2 and S3). During the initial phase of infection (1 dpi), the proportions of monocytes/macrophages (MRC1L-B^+^) in the T.T and T.F.T groups did not significantly differ from those of the NT group (Fig. S1D). However, at 3 dpi, both the proportion and the absolute number of MRC1L-B^+^ cells in the spleen were higher in the T.F.T group than in the T.T group, indicating a more substantial bacterial infection (Fig. 3F). However, during infection, both the proportion (1 and 3 dpi) and the absolute number (1 dpi) of γδ T cells in the cecum lamina propria were higher in the T.T group than in the control group. Similar changes were not observed in the florfenicol-treated group (Fig. 3G, Fig. S1E). Taken together, these results demonstrate that florfenicol-induced dysbiosis not only facilitates APEC colonization and systemic infection, but also hinders the immune responses crucial for effectively controlling infection both locally and systemically.

### Florfenicol-induced dysbiosis reduces butyrate production

**Fig. 4:**
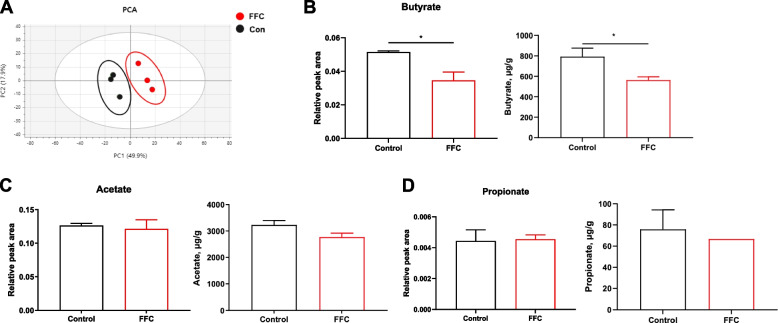
Cecal butyrate levels are reduced in florfenicol-treated chickens. **A** Alterations in cecal metabolites were identified in a principal component analysis (*n* = 3 per group) using the same scheme as in Fig. 1. **B**–**D** The relative peak area (left panel) and the concentration (right panel) of butyrate, acetate, and propionate as determined using NMR and HPLC. FFC, florfenicol. Results are presented as the mean ± SEM. Statistical differences were determined in a *t*-test; *P < 0.05

Microbiota-derived metabolites, particularly SCFAs, regulate the hypoxic environment in the gut lumen [9]. To examine whether these changes are influenced by altered gut microbiota in florfenicol-induced dysbiosis, metabolite profiles were evaluated using NMR and HPLC. As shown in Fig. 4A, florfenicol administration significantly altered the metabolite profiles in the cecal contents. Specifically, the relative amount and concentration of butyrate were significantly reduced (Fig. 4B) whereas acetate (Fig. 4C) and propionate (Fig. 4D) levels changed only slightly. These findings indicate that florfenicol treatment leads to a decrease in butyrate levels.

### Butyrate administration restores gut homeostasis impaired by florfenicol

**Fig. 5:**
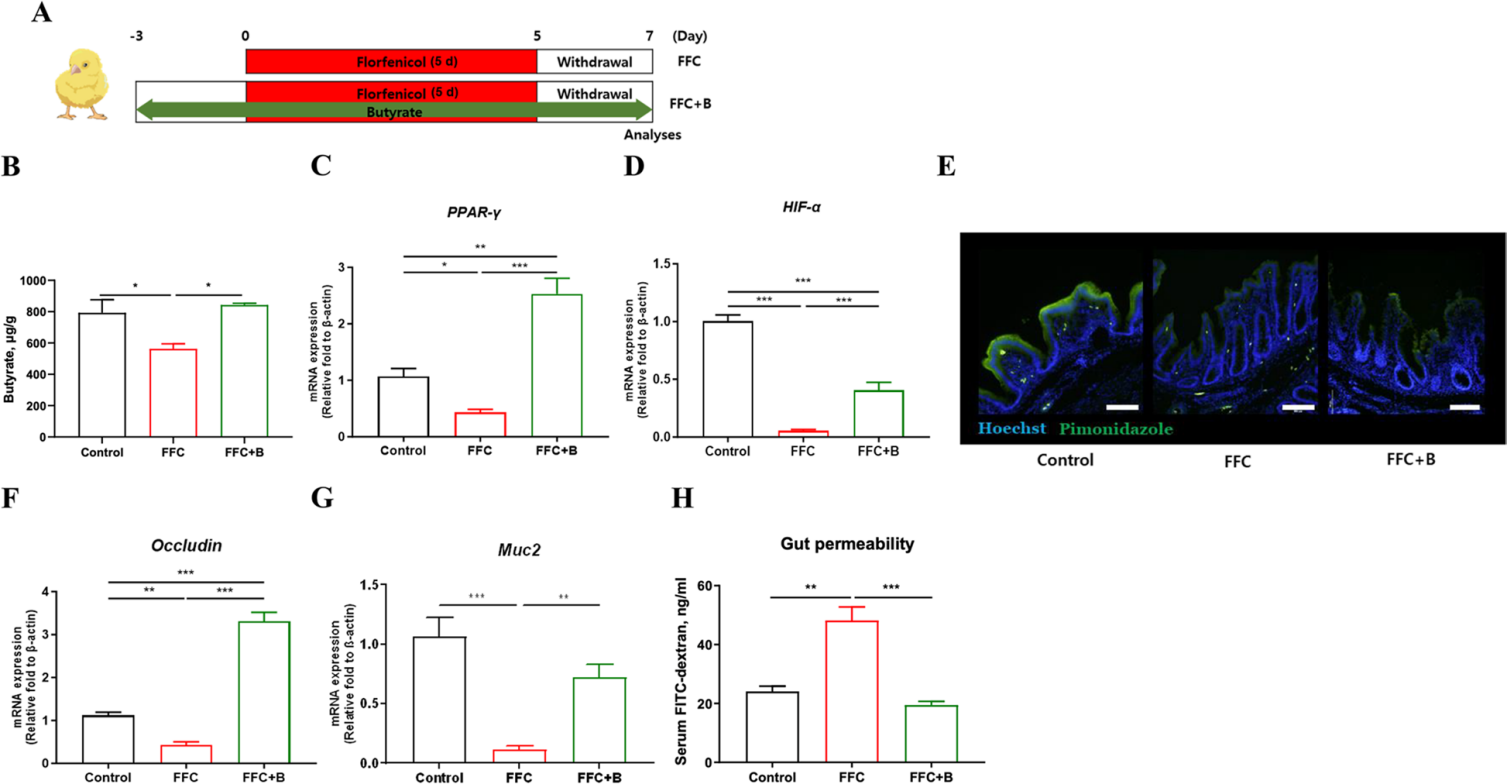
Butyrate administration in chickens with dysbiosis restores hypoxic conditions and mitigates gut permeability. **A** Prior to and throughout florfenicol administration, the chickens (*n* = 6) were supplied with butyrate in their drinking water. **B** Restoration of cecal butyrate was examined using HPLC. Following butyrate treatment, genomic changes related to hypoxia (**C** and **D**), hypoxic conditions (scale bar = 100 µm) (**E**), genomic changes related to intestinal barrier function (**F** and **G**), and changes in gut permeability (**H**) were examined. FFC, florfenicol. FFC + B, florfenicol + butyrate. Results are presented as the mean ± SEM. Statistical differences were determined in a Tukey test; *P < 0.05, **P < 0.01, ***P < 0.001

The relationship between the reduction in butyrate levels resulting from dysbiosis and overall gut integrity (Fig. 5A) was investigated by examining the effect of butyrate administration in florfenicol-treated chickens (Fig. 5B, Fig. S4A). Butyrate administration substantially restored the expression of *PPAR-γ* (Fig. 5C) and *HIF-1α* (Fig. 5D), which play key roles in the metabolism of epithelial cells under hypoxia. In addition, pimonidazole accumulation in the intestinal epithelial cells of the butyrate-treated chickens was more similar to that of the control group than the FFC group (Fig. 5E). These results indicated that butyrate administration partly restores the cecal hypoxia impaired by florfenicol treatment.

Hypoxia adversely affects cecal epithelial cells by favoring glycolysis as the predominant metabolic pathway, resulting in high levels of lactate [48] that promote *E*. *coli* growth in the intestinal lumen [49]. We therefore examined the levels of lactate in the cecal contents of chickens with florfenicol-induced dysbiosis. Elevated lactate levels were associated with a rise in potentially harmful bacteria (Fig. S4C), an effect that was mitigated by butyrate administration (Fig. S4B).

The potential of butyrate to restore gut integrity impaired by florfenicol-induced dysbiosis was investigated by examining the tight junctions of intestinal epithelial cells and mucin production. Butyrate administration increased the expression of Occludin (Fig. 5F) and *Muc2* (Fig. 5G) and reduced the gut permeability (Fig. 5H) resulting from florfenicol-induced dysbiosis. Collectively, the results suggested that by restoring gut integrity and the hypoxic environment of the intestinal lumen, butyrate restores cecal homeostasis.

### Butyrate administration reduces the susceptibility to systemic APEC infection

**Fig. 6:**
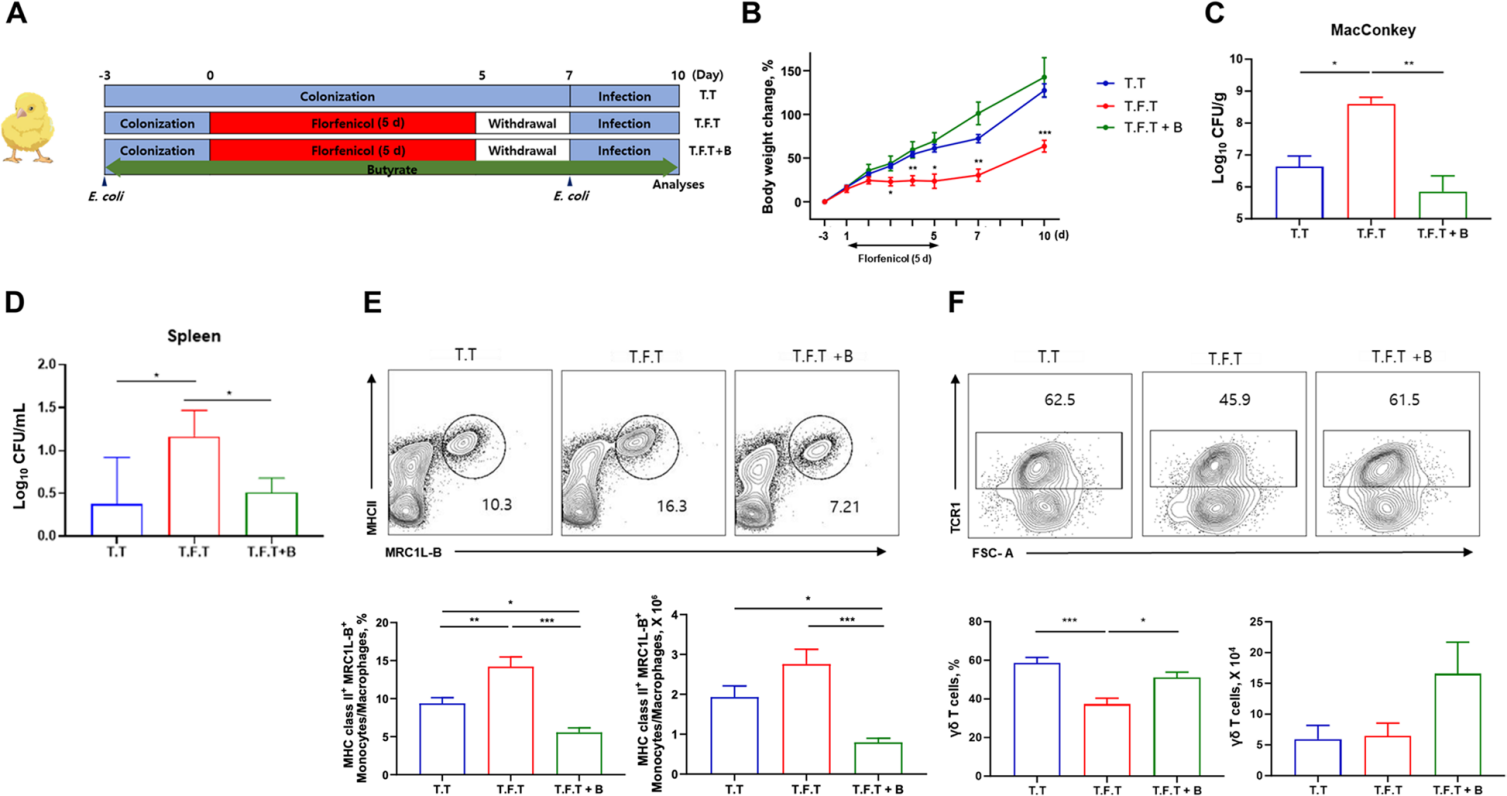
Butyrate administration alleviates systemic APEC infection and reverses the immune cell changes in chickens. **A** Butyrate was administered in the chickens’ drinking water starting with the first APEC infection and continued for 3 d after the second APEC infection. **B–F** Body weight changes (**B**), the presence of pathobionts in the cecal contents (**C**), the mean log_10_ CFU/mL of APEC in the spleen (**D**), and changes in the percentage and absolute number of splenic macrophages (**E**) and lamina propria γδ T cells (**F**) were evaluated. T.T, APEC double infection without florfenicol treatment. T.F.T, APEC double infection with florfenicol treatment. T.F.T + B APEC double infection with florfenicol and butyrate treatment. Statistical differences were determined in a Tukey test; *P < 0.05, **P < 0.01, ***P < 0.001

Given the ability of butyrate to restore hypoxia and the integrity of tight junctions in the intestines of chickens with florfenicol-induced dysbiosis, its ability to restore resistance to APEC infection was also examined (Fig. 6A). The administration of butyrate (T.F.T + B) resulted in an increase in body weight (Fig. 6B) and a decrease in the colonization of pathobionts in the cecum by 3 dpi (Fig. 6C). It also decreased systemic APEC infection, as determined based on the presence of APEC in the spleen (Fig. 6D). However, at 1 dpi, and thus during the early stage after infection, APEC was not detected in the spleens of any of the groups (Fig. S5A).

The T.F.T + B group showed lower percentage and absolute number of MRC1L-B^+^ cells in the spleen as compared to the T.T group at 1 and 3 dpi (Fig. S5B, Fig. 6E). Cecum lamina propria showed a restoration of both the proportion and absolute number of γδ T cells to normal levels in chickens treated with butyrate treatment at 1 and 3 dpi (Fig. S5C, Fig. 6F).

These results showed that butyrate treatment effectively reestablishes the local and systemic immunological profiles altered by dysbiosis. Specifically, butyrate counteracts the adverse effects of florfenicol, by restoring body weight and reducing the colonization of pathobionts as well as the occurrence of systemic APEC infection. It also restores MRC1L-B^+^ cells in the spleen and normalizes the proportion of γδ T cells in the cecum lamina propria.

### Correlation between the differential gut microbiota and metabolites

**Fig. 7:**
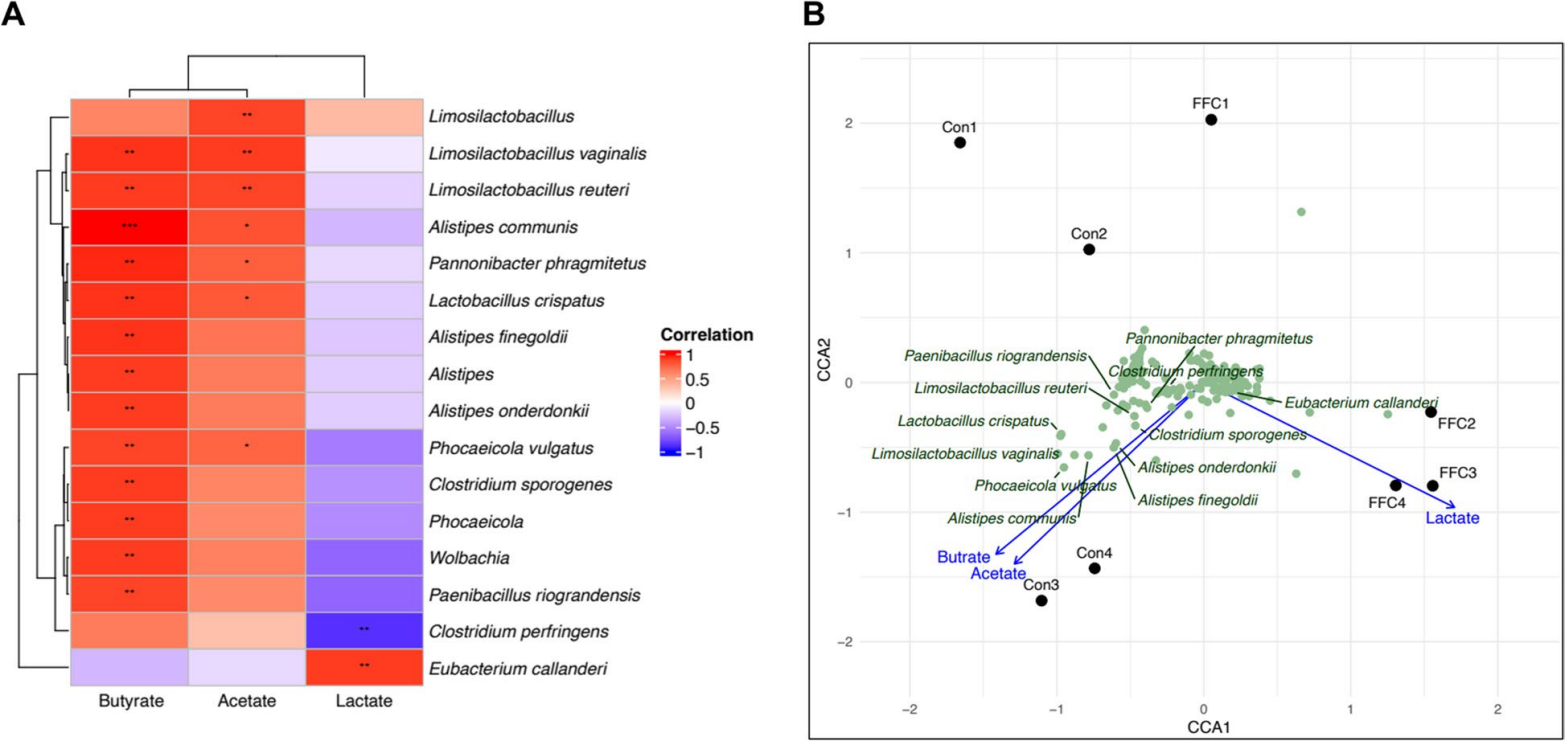
Correlation between key bacterial taxa and differential metabolites. **A** Spearman correlations between differential metabolites and bacterial taxa in the FFC and control groups. Positive correlations are shown in red, and negative correlations in blue. **B** CCA of differential metabolites and bacterial taxa for the FFC and control groups, illustrating the correlation between bacterial community structures and metabolite factors. Arrows represent the direction and magnitude of correlations between metabolite factors and key bacterial taxa. Statistical differences were tested using the cor.test function, and significance levels are indicated as follows: *P < 0.05, **P < 0.01, ***P < 0.001

After observing marked differences in both metabolite levels and microbial compositions following florfenicol treatment, we conducted pairwise Spearman rank correlation (Fig. 7A) and CCA (Fig. 7B) to determine key associations among microbes and metabolites. In particular, three metabolites (butyrate, acetate, and lactate) were analyzed, while propionate was excluded from the correlation analysis due to its extremely low detected levels. Among the 338 major microbial species included, Spearman correlation analysis revealed that butyrate clustered closely with acetate and showed strong positive correlations with *Limosilactobacillus vaginalis, Limosilactobacillus reuteri, Alistipes communis, Pannonibacter phragmitetus, Lactobacillus crispatus, Alistipes finegoldii, Alistipes* spp., *Alistipes onderdonkii, Phocaeicola vulgatus, Clostridium sporogenes, Phocaeicola *spp., and *Wolbachia*. Notably, a subset of these species—*L. vaginalis, L. reuteri, A. communis, P. phragmitetus, L. crispatus,* and *P. vulgatus*—also exhibited positive correlations with acetate. In contrast, lactate showed an opposing pattern relative to butyrate and acetate, displaying a positive correlation with *Eubacterium callanderi,* while exhibiting a negative correlation with *Clostridium perfringens* (Fig. 7A).

Consistent with the Spearman correlation results, the CCA ordination analysis further highlighted the clear separation of butyrate and acetate from lactate. Specifically, butyrate and acetate were more pronounced in the control group, whereas lactate was elevated in the florfenicol group. Moreover, *Eubacterium callanderi* displayed a strong association with lactate in the florfenicol group, whereas in the control group, butyrate and acetate were closely linked to the microbiota that showed high correlation in the Spearman analysis (Fig. 7B).

## Discussion

Florfenicol is a synthetic antibiotic classified within the amphenicol group, which includes chloramphenicol, thiamphenicol, and azidamfenicol, all characterized by their phenylpropanoid structure. It was specifically developed for veterinary use to overcome the limitations of chloramphenicol, which is banned for use in food-producing animals due to its toxic side effects in humans. Florfenicol is effective against a wide range of pathogenic bacteria, including both aerobic and anaerobic as well as Gram-positive and Gram-negative species. It is typically administered to poultry via drinking water and is noted for its efficacy in treating respiratory infections caused by *E*. *coli*, *Pasteurella* spp., and *Haemophilus* spp.

Many antibiotics are used as antibiotic growth promoters (AGPs) and in disease prevention in the poultry industry; however, florfenicol, as a relatively new antibiotic first introduced in Japan in 1990, has never been used as an AGP in poultry production. Recent studies have reported that the combined *in ovo* administration of florfenicol with probiotics significantly improves growth performance and enhances resistance to pathogenic infections [50]. Thus, despite the current ban on the use of antibiotics for AGP and disease prevention in many countries, due to antimicrobial resistance concerns, florfenicol may have beneficial effects in poultry when used appropriately.

The detrimental impacts of antibiotics on gut microbiota dysbiosis have been well-documented in both human and murine studies, including the disruption of intestinal homeostasis and the exacerbation of inflammatory responses. Host–gut microbiota interactions are crucial for maintaining intestinal homeostasis [51], with commensal microbiota playing a key role in reducing the risk of pathogen infection, particularly through the production of metabolites [52]. Among these metabolites, SCFAs (such as butyrate) play a central role in regulating intestinal epithelial function. Butyrate contributes to maintaining epithelial hypoxia by activating PPAR-γ, which enhances mitochondrial β-oxidation and oxygen consumption, thereby preserving the anaerobic environment in the intestinal lumen [53]. Conversely, decreased butyrate levels disrupt this hypoxic state, leading to increased oxygen availability at the mucosal surface, which favors the expansion of facultative anaerobic pathogens [54]. This shift in microbial composition has been linked to increased pathogen burden and further dysregulation of gut homeostasis.

In this study, we observed that antibiotic-induced dysbiosis disrupts the homeostatic environment of the intestine, leading to decreased expression of key factors such as HIF-1α and PPAR-γ. Previous studies have reported that these factors can be regulated by various metabolites, among which SCFAs have been identified as the most promising candidates. In addition to SCFAs, lactate is another abundant metabolite in the dysbiotic gut environment, and mouse studies have suggested that it may serve as a favorable metabolite for pathogenic bacterial growth. Based on this evidence, we selected SCFAs and their function as the primary focus of investigation in chickens to explore species-specific responses and potential implications for poultry health. Our findings revealed that among SCFAs, butyrate plays a key role in the recovery of hypoxia-related pathways and gut permeability. Additionally, butyrate and acetate showed a positive correlation with normal microbiota, whereas lactate was associated with dysbiosis-derived microbiota. However, beyond these metabolites, various other metabolic compounds may also influence intestinal epithelial cell metabolism and contribute to the changes in the gut microenvironment. Therefore, further studies are needed to explore the roles of other metabolites in chickens and their impact on gut homeostasis.

As noted above, the changes in metabolites and intestinal inflammatory responses caused by antibiotics-induced dysbiosis have been studied [55] mostly in mice and humans, with relatively little research in poultry. In mice, prophylactic antibiotic treatment was shown to impair resistance against bacterial and fungal infections [48, 56]. A similar study in chickens reported that the administration of prophylactic antibiotic increased susceptibility to *Salmonella* infection, linked to alterations in the gut microbiota and the reduced expression of tight junction proteins [57]. This study also examined the changes in cecal metabolomes and identified linoleic acid as an important metabolite. The effects of prophylactic antibiotic administration in the early versus the later life of chickens have also been compared [30]. Prophylactic antibiotic treatment during early life was shown to increase the *Escherichia*-*Shigella* population. Following H9N2 AIV infection, larger disturbances in the composition of the gut microbiota in these chickens than in chickens with short-term antibiotic treatment later in life were observed.

**Fig. 8:**
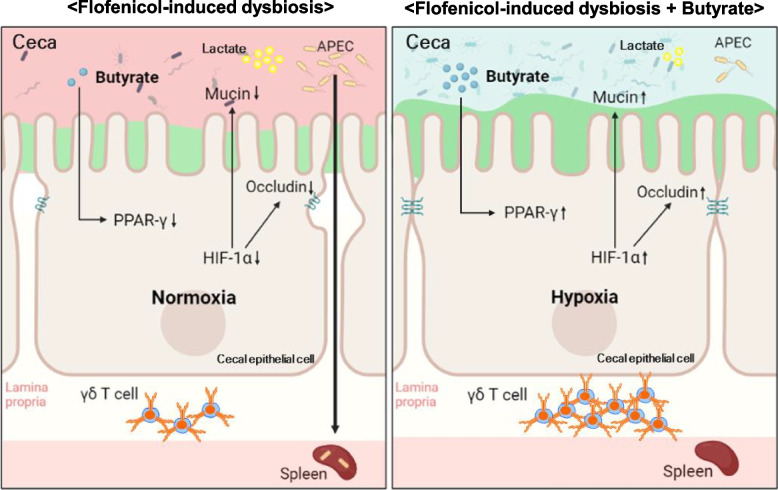
Florfenicol-induced dysbiosis disrupts cecal homeostasis and compromises disease resistance in chickens. Florfenicol-induced dysbiosis impairs cecal homeostasis by reducing butyrate levels, which increased the susceptibility to APEC infection (left). By restoring homeostasis, butyrate administration reduces the risk of APEC infection (right)

However, to the best of our knowledge, ours is the first study to investigate the effects of antibiotics on bacterial infection susceptibility in chickens, with a focus on the mechanisms related to gut microbiota-host interactions, alterations in the intestinal environment, and the impacts on the immune system (Fig. 8). Our results contribute to filling a significant gap in our understanding of the effects of antibiotics use in chickens. Nonetheless, improving the health and disease resistance of avian species requires further investigations of gut microbiota -host interactions and their relationship to the immune system.

Florfenicol is commonly used to control APEC and *Salmonella* spp. In this study, it was administered over 5 d, followed by a 2-d withdrawal period, in accordance with standard industrial treatment protocols [35]. This regimen led to a decrease in intestinal butyrate levels that in turn compromised the maintenance of intestinal hypoxia by inducing PPAR-γ expression and facilitating β-oxidation in intestinal epithelial cells. Butyrate treatment in chickens increased PPAR-γ expression, but was insufficient to fully restore cecal hypoxia, evidenced by the incomplete restoration of HIF-1α transcript levels, suggesting that PPAR-γ alone does not maintain intestinal hypoxia. Florfenicol-induced dysbiosis also decreased the expression of Occludin and *Muc2*, two genes associated with intestinal barrier function. In a preliminary study, we evaluated the expression of the tight junction proteins claudin-1, claudin-2, claudin-3, and zona occludens-1 (*ZO-1*) during the withdrawal period, but the differences in their expression were not significant (Fig. S6). While no differences were observed during dysbiosis, such differences may emerge in a dysbiosis-induced infection state. A limitation of this study is the lack of analysis for other tight junction proteins under infection conditions.

The regulation of hypoxic conditions in the gut is influenced by gut microbiota as well as host factors. Antibiotics reduce microbial diversity and abundance, increasing oxygen levels in the gut [58]. The resulting promotion in the growth of aerobic bacteria can create a pathological state and induce epithelial damage [48]. From the host's perspective, changes in host cellular metabolism may alter hypoxic conditions within the intestinal lumen, as occurs following a shift from mitochondrial oxidative phosphorylation, which consumes significant amounts of oxygen, to glycolysis, which increases oxygen levels [59]. Further studies are needed to identify additional factors that may contribute to the inadequate restoration of hypoxia with butyrate treatment. These factors include shifts in oxygen-utilizing bacteria and changes in the metabolic activity of intestinal epithelial cells.

APEC causes enormous economic losses in the poultry industry [2]. The APEC strain used in this study harbors the K1 antigen and is able to replicate within macrophages. Moreover, as this strain can be transmitted from chickens to humans, it poses a public health threat. APEC can survive in dry environments and be transmitted through the air, causing respiratory infections in chickens [60, 61]. However, APEC is also an opportunistic pathogen in the gut that can cause systemic infections when gut homeostasis is disrupted [62]. Despite this, there is limited research on how APEC, as an opportunistic pathogen in the gut, causes systemic infections in chickens. The present study indicated that APEC infection had no effect on morbidity or mortality in chickens (data not shown), suggesting that, while APEC can persist in the gut without causing disease, it may induce disease if the barrier function is compromised. Additionally, this study also demonstrated that florfenicol-induced dysbiosis can promote APEC systemic infection by weakening the intestinal barrier through reduced butyrate levels. Despite our findings, research on the mechanisms of APEC infection through the gut in chickens is limited on both the bacterial and the host perspectives.

Florfenicol-induced dysbiosis also exacerbated systemic APEC infection, which was associated with an increase in monocyte/macrophage lineage cells in the spleen. However, this study did not determine whether APEC’s increased systemic infection was primarily attributable to increased gut permeability or was also related to alterations in the systemic immune system. The spleen, as a lymphoid organ, has various subsets of immune cells in discrete sites [63], each with distinct role during bacterial infections [64]. While research on this topic in poultry is limited, existing studies have characterized the various immune cell subsets and their roles in the spleen [65, 66]. In previous work, we characterized the subsets of monocyte/macrophage lineage cells in the spleen based on their patterns of MRC1L-B and MHCII expression and determined the role of each subset [37]. In the current study, there were no differences in the proportions of the different subsets of splenic monocyte/macrophage lineage cells (data not shown). Previous studies of APEC infection in chickens reported an increase in the levels of proteins in phagosome and lysosome pathways, which are crucial for pathogen clearance, such as Sec61, vATPase, and cathepsin, and in the expression of the respective genes [67]. Whether florfenicol-induced dysbiosis in chickens affects these pathways should be addressed in further studies.

Our evaluation of the immune responses against APEC, an opportunistic gut pathogen, focused on changes in immune cells in the cecal lamina propria, a site often overlooked in poultry research, and revealed a decrease in γδ T cells. In poultry, γδ T cells are divided into TCR1^+^CD8^−^ , CD8αα^+^ and CD8αβ^+^ subsets [68, 69], although which subset produces IFN-γ and IL-17 is unknown. The influence of SCFAs, such as butyrate, on the gastrointestinal immune system has been shown to involve the regulation of γδ T cell function through a histone deacetylase-dependent mechanism [70, 71]. In our study, butyrate treatment restored the reduction of γδ T cells associated with florfenicol-induced dysbiosis in laying hens infected with APEC, but without affecting the proportions of CD8αα^+^ and CD8αβ^+^ γδ T cell subsets. Further research is needed to explore the effects of butyrate on activation and function of γδ T cells in APEC-infected chickens. Aside from γδ T cells, other immune cells that functionally respond to SCFAs include Tregs, macrophages, and CD8^+^ T cells [72, 73]. In a previous study we investigated the gut microbiota-host relationship in germ-free chickens treated with broad-spectrum antibiotics [74]. Among the examined SCFAs, the decrease in propionate was found to be associated with a reduction in CD4^+^CD25^+^ T cells in the cecal tonsil. This cell population contains Foxp3^+^ cells, whose function is similar to that of Tregs in mice [75]. Therefore, changes in CD4^+^CD25^+^ T cells in response to antibiotic-induced reductions in SCFAs merit further study.

Early-life dysbiosis in chickens, such as triggered by antibiotics exposure or dietary factors, can have profound and lasting health impacts. The composition of the gut microbiota during development plays a crucial role in shaping long-term health outcomes, including metabolic function [76, 77], immune responses [78], productivity [79], and overall longevity [80]. For instance, studies in mice have shown that early-life antibiotics exposure disrupts the production of key metabolites, such as indole-3-propionic acid, leading to increases in allergic and inflammatory conditions in adulthood [81]. Furthermore, dysbiosis during early life can exacerbate the effects of a high-fat diet in adulthood, resulting in severe metabolic dysregulation [82]. In poultry research, regulation of the gut microbiota through fecal transplantation in young birds has been shown to influence behavior, serotonergic activity, stress response, innate immunity, and cecal microbiota composition in adulthood [83]. These findings underscore the importance of maintaining a balanced gut microbiota during early life to prevent long-term health complications. Furthermore, infections at a young age can impair the development of various immune cells, with continued effects into adulthood [84] and more severe illness following reinfection [85]. A better understanding of the impact of early-life gut dysbiosis on the adult immune system and on reinfection responses awaits additional studies.

## Conclusion

In conclusion, this study investigated the impact of florfenicol-induced dysbiosis on APEC infection in chicken, with a specific focus on the underlying mechanisms that influence the interactions between gut microbiota and host’s immune responses. Our observation revealed that florfenicol treatment results in a reduction in butyrate levels, impairing intestinal hypoxia and might exacerbate APEC infection by compromising the gut barrier integrity. Despite increased *PPAR-γ* expression with butyrate treatment, full restoration of intestinal hypoxia was not achieved, highlighting the intricate regulation of gut hypoxia involving both microbial and host factors. We also found that dysbiosis resulted in increased monocyte/macrophage lineage cells in the spleen. However, it remains uncertain whether this increase is solely caused by increased gut permeability or by systemic immunological changes. Furthermore, there was a reduction in γδ T cells in the cecal lamina propria, underscoring the importance of γδ T cells in the early phases of infection and their role in bacterial clearance and immune modulation. Our findings suggest that while SCFAs, such as butyrate, influence immune cell activity, further studies are needed to investigate how they specifically affect the function of γδ T cells and the broader implications of antibiotic-induced dysbiosis on the health and disease resistance in chickens. Gaining a comprehensive understanding of these mechanisms could offer valuable insights into the ways dysbiosis influences systemic infections and help in the development of strategies to mitigate the adverse effects of antibiotics on poultry health.

## Supplementary Information

Additional file1: Fig. S1. Florfenicol-induced dysbiosis increases susceptibility to systemic APEC infection. Chickens (*n* = 6) were infected with APEC 3 d prior to florfenicol treatment. Following the withdrawal phase, they were re-infected with APEC. Systemic infection at 1 dpi was quantified by determining (A) the mean log_10_ CFU/mL in the spleen and the levels of (B) *wzx* and (C) *neuC1* mRNA in bacteria isolated from APEC O1:K1, *E. coli* K88 and K99, and *Salmonella* Enteritidis. Changes in the percentage and absolute number of (D) splenic macrophages and (E) lamina propria γδ T cells. NT, non-treated. T.T, APEC double infection without florfenicol treatment. T.F.T, APEC double infection with florfenicol treatment. Statistical differences were determined in a Tukey test; *P < 0.05, **P < 0.01, ***P < 0.001.

Additional file 2: Fig. S2. Gating strategy for splenic monocytes/macrophages. Live single splenocytes were gated by FSC-A vs. SSC-A based on CD45 expression, followed by the gating of monocytes/macrophages based on MHC class II and MRC1L-B expression.

Additional file 3: Fig. S3. Gating strategy T cells in the lamina propria. Live single cells were gated by FSC-A vs. SSC-A. Single cells from lamina propria were gated based on CD45 expression , followed by CD3 to identify T cells. T cells were divided into γδ T cells and non-γδ T cells based on TCR γδ (TCR1) expression, with γδ T cells then sub-divided into three subpopulations. Non-γδ T cells were sub-divided based on CD4 expression. CD4^+^ and CD4^–^ T cells were then sub-divided into subpopulations based on CD8a or CD8b expression.

Additional file 4: Fig. S4. Butyrate administration restores gut homeostasis impaired by florfenicol treatment. To achieve butyrate levels similar to those under homeostasis conditions in the presence of florfenicol-induced dysbiosis, chickens were provided with 50 mmol/L or 100 mmol/L butyrate in their drinking water. (A) Butyrate levels in the cecum were then measured. (B) Alteration of cecal lactate levels measured by HPLC. (C) Pathobionts in the cecal content from control, florfenicol, and florfenicol+butyrate groups as measured based on CFU counts on MacConkey agar. FFC, florfenicol. FFC+B, florfenicol+butyrate. Statistical differences were determined in a Tukey test; *P < 0.05, **P < 0.01, ***P < 0.001.

Additional file 5: Fig. S5. Butyrate administration restores the susceptibility to systemic APEC infection. Butyrate was administered to chickens in their drinking water starting with the first APEC infection until 3 d after re-infection. Systemic infection of APEC was quantified at 1 dpi by determining the (A) mean log_10_ CFU/mL in the spleen and the changes in the percentage and absolute number of (B) splenic macrophages and (C) lamina propria γδ T cells. T.T, APEC double infection without florfenicol treatment. T.F.T, APEC double infection with florfenicol treatment. T.F.T+B APEC double infection with florfenicol and butyrate treatment. Statistical differences were determined in a Tukey test; *P < 0.05, ***P < 0.001.

Additional file 6: Fig. S6. Claudin-1, -2, -3, and ZO-1 are not affected by florfenicol treatment. The mRNA expression levels of Claudin-1, -2, -3, and* ZO-1* in chickens treated with PBS or florfenicol. FFC, florfenicol. Results are presented as the mean ± SEM.

## Data Availability

The datasets used and analyzed in the current study are available from corresponding authors on reasonable request.

## Abbreviations

APEC: Avian pathogenic *E. coli*
cDNA: Complementary deoxyribonucleic acid
CFU: Colony forming unit
EDTA: Ethylenediaminetetraacetic acid
ExPEC: Extraintestinal pathogenic *E. coli*
FBS: Fetal bovine serum
FFC: Florfenicol
FITC: Fluorescein isothiocyanate
HBSS: Hanks’ balanced salt solution
HEPES: Hydroxyethyl piperazine ethane sulfonic acid
HIF: Hypoxia-inducible factor
HPLC: High performance liquid chromatography
IEL: Intraepithelial lymphocyte
LPL: Lamina propria lymphocyte
NMR: Nuclear magnetic resonance
PBS: Phosphate buffered saline
PCA: Principal component analysis
PCR: Polymerase chain reaction
PPAR: Peroxisome proliferator-activated receptor
RNA: Ribonucleic acid
SCFA: Short-chain fatty acid
Treg: Regulatory T cell

## References

[1] Desvaux M, Dalmasso G, Beyrouthy R, Barnich N, Delmas J, Bonnet R. Pathogenicity factors of genomic islands in intestinal and extraintestinal *Escherichia coli*. Front Microbiol. 2020;11:2065. 33101219 10.3389/fmicb.2020.02065 PMC7545054

[2] Apostolakos I, Laconi A, Mughini-Gras L, Yapicier OS, Piccirillo A. Occurrence of colibacillosis in broilers and its relationship with avian pathogenic *Escherichia coli* (APEC) population structure and molecular characteristics. Front Vet Sci. 2021;8:737720. 34568479 10.3389/fvets.2021.737720 PMC8456121

[3] Horn F, Correa AM, Barbieri NL, Glodde S, Weyrauch KD, Kaspers B, et al. Infections with avian pathogenic and fecal *Escherichia coli* strains display similar lung histopathology and macrophage apoptosis. PLoS ONE. 2012;7(7):e41031. 22848424 10.1371/journal.pone.0041031 PMC3405075

[4] Martin MD, Skon-Hegg C, Kim CY, Xu J, Kucaba TA, Swanson W, et al. CD115^+^ monocytes protect microbially experienced mice against *E. coli*-induced sepsis. Cell Rep. 2023;42(11):113345. 38111515 10.1016/j.celrep.2023.113345 PMC10727454

[5] de Brito BG, Gaziri LCJ, Vidotto MC. Virulence factors and clonal relationships among strains isolated from broiler chickens with cellulitis. Infect Immun. 2003;71(7):4175–7. 12819112 10.1128/IAI.71.7.4175-4177.2003 PMC162012

[6] Duszka K, Oresic M, Le May C, Konig J, Wahli W. PPARγ modulates long chain fatty acid processing in the intestinal epithelium. Int J Mol Sci. 2017;18(12):2559. 10.3390/ijms18122559 PMC5751162 29182565

[7] Das NK, Schwartz AJ, Barthel G, Inohara N, Liu Q, Sankar A, et al. Microbial metabolite signaling is required for systemic iron homeostasis. Cell Metab. 2020;31(1):115. 31708445 10.1016/j.cmet.2019.10.005 PMC6949377

[8] Cani PD, Van Hul M, Lefort C, Depommier C, Rastelli M, Everard A. Microbial regulation of organismal energy homeostasis. Nat Metab. 2019;1(1):34–46. 32694818 10.1038/s42255-018-0017-4

[9] Pral LP, Fachi JL, Corrêa RO, Colonna M, Vinolo MAR. Hypoxia and HIF-1 as key regulators of gut microbiota and host interactions. Trends Immunol. 2021;42(7):604–21. 34171295 10.1016/j.it.2021.05.004 PMC8283795

[10] Arpaia N, Campbell C, Fan X, Dikiy S, van der Veeken J, deRoos P, et al. Metabolites produced by commensal bacteria promote peripheral regulatory T-cell generation. Nature. 2013;504(7480):451–5. 24226773 10.1038/nature12726 PMC3869884

[11] Chang PV, Hao L, Offermanns S, Medzhitov R. The microbial metabolite butyrate regulates intestinal macrophage function via histone deacetylase inhibition. Proc Natl Acad Sci U S A. 2014;111(6):2247–52. 24390544 10.1073/pnas.1322269111 PMC3926023

[12] Buret AG, Motta JP, Allain T, Ferraz J, Wallace JL. Pathobiont release from dysbiotic gut microbiota biofilms in intestinal inflammatory diseases: a role for iron? J Biomed Sci. 2019;26(1):1. 30602371 10.1186/s12929-018-0495-4 PMC6317250

[13] Le Roy CI, Woodward MJ, Ellis RJ, La Ragione RM, Claus SP. Antibiotic treatment triggers gut dysbiosis and modulates metabolism in a chicken model of gastro-intestinal infection. BMC Vet Res. 2019;15:37. 10.1186/s12917-018-1761-0 PMC6347850 30683093

[14] Winter SE, Baumler AJ. Gut dysbiosis: Ecological causes and causative effects on human disease. Proc Natl Acad Sci U S A. 2023;120(50):e2316579120. 38048456 10.1073/pnas.2316579120 PMC10722970

[15] Kriss M, Hazleton KZ, Nusbacher NM, Martin CG, Lozupone CA. Low diversity gut microbiota dysbiosis: drivers, functional implications and recovery. Curr Opin Microbiol. 2018;44:34–40. 30036705 10.1016/j.mib.2018.07.003 PMC6435260

[16] Feng Y, Huang Y, Wang Y, Wang P, Song H, Wang F. Antibiotics induced intestinal tight junction barrier dysfunction is associated with microbiota dysbiosis, activated NLRP3 inflammasome and autophagy. PLoS ONE. 2019;14(6):e0218384. 31211803 10.1371/journal.pone.0218384 PMC6581431

[17] Dissanayake WMN, Chandanee MR, Lee SM, Heo JM, Yi YJ. Change in intestinal alkaline phosphatase activity is a hallmark of antibiotic-induced intestinal dysbiosis. Anim Biosci. 2023;36(9):1403–13. 37170509 10.5713/ab.23.0052 PMC10472154

[18] Zha X, Su S, Wu D, Zhang P, Wei Y, Fan S, et al. The impact of gut microbiota changes on the intestinal mucus barrier in burned mice: a study using 16S rRNA and metagenomic sequencing. Burns Trauma. 2023;11:tkad056. 38130728 10.1093/burnst/tkad056 PMC10734567

[19] Aguilera M, Cerda-Cuellar M, Martinez V. Antibiotic-induced dysbiosis alters host-bacterial interactions and leads to colonic sensory and motor changes in mice. Gut Microbes. 2015;6(1):10–23. 25531553 10.4161/19490976.2014.990790 PMC4615720

[20] Shi Y, Kellingray L, Zhai Q, Gall GL, Narbad A, Zhao J, et al. Structural and functional alterations in the microbial community and immunological consequences in a mouse model of antibiotic-induced dysbiosis. Front Microbiol. 2018;9:1948. 30186263 10.3389/fmicb.2018.01948 PMC6110884

[21] Xu C, Ruan B, Jiang Y, Xue T, Wang Z, Lu H, et al. Antibiotics-induced gut microbiota dysbiosis promotes tumor initiation via affecting APC-Th1 development in mice. Biochem Biophys Res Commun. 2017;488(2):418–24. 28506830 10.1016/j.bbrc.2017.05.071

[22] Jin S, Zhao D, Cai C, Song D, Shen J, Xu A, et al. Low-dose penicillin exposure in early life decreases Th17 and the susceptibility to DSS colitis in mice through gut microbiota modification. Sci Rep. 2017;7:43662. 28272549 10.1038/srep43662 PMC5341569

[23] He B, Liu Y, Hoang TK, Tian X, Taylor CM, Luo M, et al. Antibiotic-modulated microbiome suppresses lethal inflammation and prolongs lifespan in Treg-deficient mice. Microbiome. 2019;7:145. 31699146 10.1186/s40168-019-0751-1 PMC6839243

[24] Gao W, Liu X, Zhang S, Wang J, Qiu B, Shao J, et al. Alterations in gut microbiota and inflammatory cytokines after administration of antibiotics in mice. Microbiol Spectr. 2024;12(8):e0309523. 38899904 10.1128/spectrum.03095-23 PMC11302321

[25] Ye Y, Tong HYK, Chong WH, Li Z, Tam PKH, Baptista-Hon DT, et al. A systematic review and meta-analysis of the effects of long-term antibiotic use on cognitive outcomes. Sci Rep. 2024;14:4026. 38369574 10.1038/s41598-024-54553-4 PMC10874946

[26] Lohani M, Ahmad AH, Singh KP, Verma S. Pharmacokinetics and residual studies of florfenicol following multiple dose oral administration in poultry. J Appl Anim Res. 2010;38(1):9–12.

[27] Li X, Chen L, Yue H, Feng H, Xu E, Wei X, et al. Depletion of florfenicol and florfenicol amine in eggs of laying hens and growing pullets after oral administration. Food Addit Contam Part A Chem Anal Control Expo Risk Assess. 2020;37(9):1449–58. 32619394 10.1080/19440049.2020.1769196

[28] Li P, Zhu T, Zhou D, Lu W, Liu H, Sun Z, et al. Analysis of resistance to florfenicol and the related mechanism of dissemination in different animal-derived bacteria. Front Cell Infect Microbiol. 2020;10:369. 32903722 10.3389/fcimb.2020.00369 PMC7438884

[29] Van Cuong N, Kiet BT, Phu DH, Van NTB, Hien VB, Thwaites G, et al. Effects of prophylactic and therapeutic antimicrobial uses in small-scale chicken flocks. Zoonoses Public Health. 2021;68(5):483–92. 33934522 10.1111/zph.12839 PMC8573609

[30] Liang X, Zhang Z, Wang H, Lu X, Li W, Lu H, et al. Early-life prophylactic antibiotic treatment disturbs the stability of the gut microbiota and increases susceptibility to H9N2 AIV in chicks. Microbiome. 2023;11:163. 10.1186/s40168-023-01609-8 PMC10369819 37496083

[31] Hassanin O, Abdallah F, Awad A. Effects of florfenicol on the immune responses and the interferoninducible genes in broiler chickens under the impact of *E. coli* infection. Vet Res Commun. 2014;38(1):51–8. 10.1007/s11259-013-9585-7 24254460

[32] Wang X, Han C, Cui YQ, Li SY, Jin GZ, Shi WY, et al. Florfenicol causes excessive lipid peroxidation and apoptosis induced renal injury in broilers. Ecotox Environ Safe. 2021;207:111282. 10.1016/j.ecoenv.2020.111282 32949928

[33] Hu D, Meng F, Cui Y, Yin M, Ning H, Yin Z, et al. Growth and cardiovascular development are repressed by florfenicol exposure in early chicken embryos. Poult Sci. 2020;99(5):2736–45. 32359611 10.1016/j.psj.2020.01.007 PMC7597441

[34] AL-Shahrani S, Naidoo V. Florfenicol induces early embryonic death in eggs collected from treated hens. BMC Vet Res. 2015;11:213. 10.1186/s12917-015-0536-0 PMC4538914 26282556

[35] Imran M, Fazal EH, Tawab A, Rauf W, Rahman M, Khan QM, et al. LC-MS/MS based method development for the analysis of florfenicol and its application to estimate relative distribution in various tissues of broiler chicken. J Chromatogr B Analyt Technol Biomed Life Sci. 2017;1063:163–73. 28866358 10.1016/j.jchromb.2017.08.029

[36] Nuchchanart W, Pikoolkhao P, Saengthongpinit C. Development of a lateral flow dipstick test for the detection of 4 strains of *Salmonella* spp. in animal products and animal production environmental samples based on loop-mediated isothermal amplification. Anim Biosci. 2023;36(4):654–70. 10.5713/ab.22.0151 PMC9996269 36108678

[37] Yu K, Gu MJ, Pyung YJ, Song KD, Park TS, Han SH, et al. Characterization of splenic MRC1^hi ^MHCII^lo^ and MRC1^lo^ MHCII^hi^ cells from the monocyte/macrophage lineage of White Leghorn chickens. Vet Res. 2020;51:73. 10.1186/s13567-020-00795-9 PMC7251834 32460863

[38] Kang H. Sample size determination and power analysis using the G*Power software. J Educ Eval Health Prof. 2021;18:17. 34325496 10.3352/jeehp.2021.18.17 PMC8441096

[39] Ricci C, Baumgartner J, Malan L, Smuts CM. Determining sample size adequacy for animal model studies in nutrition research: limits and ethical challenges of ordinary power calculation procedures. Int J Food Sci Nutr. 2020;71(2):256–64. 31379222 10.1080/09637486.2019.1646714

[40] Arifin WN, Zahiruddin WM. Sample size calculation in animal studies using resource equation approach. Malays J Med Sci. 2017;24(5):101–5. 29386977 10.21315/mjms2017.24.5.11 PMC5772820

[41] Li CY, Liang YQ, Qiao Y. Messengers from the gut: Gut microbiota-derived metabolites on host regulation. Front Microbiol. 2022;13:863407. 35531300 10.3389/fmicb.2022.863407 PMC9073088

[42] Van Welden S, Selfridge AC, Hindryckx P. Intestinal hypoxia and hypoxia-induced signalling as therapeutic targets for IBD. Nat Rev Gastro Hepat. 2017;14(10):596–611. 10.1038/nrgastro.2017.101 28853446

[43] Sato K, Fukao K, Seki Y, Akiba Y. Expression of the chicken peroxisome proliferator-activated receptor-gamma gene is influenced by aging, nutrition, and agonist administration. Poult Sci. 2004;83(8):1342–7. 15339009 10.1093/ps/83.8.1342

[44] Rademakers SE, Lok J, van der Kogel AJ, Bussink J, Kaanders JH. Metabolic markers in relation to hypoxia; staining patterns and colocalization of pimonidazole, HIF-1alpha, CAIX, LDH-5, GLUT-1, MCT1 and MCT4. BMC Cancer. 2011;11:167. 21569415 10.1186/1471-2407-11-167 PMC3115911

[45] Ogasawara N, Kojima T, Go M, Ohkuni T, Koizumi J, Kamekura R, et al. PPARgamma agonists upregulate the barrier function of tight junctions via a PKC pathway in human nasal epithelial cells. Pharmacol Res. 2010;61(6):489–98. 20227502 10.1016/j.phrs.2010.03.002

[46] Saeedi BJ, Kao DJ, Kitzenberg DA, Dobrinskikh E, Schwisow KD, Masterson JC, et al. HIF-dependent regulation of claudin-1 is central to intestinal epithelial tight junction integrity. Mol Biol Cell. 2015;26(12):2252–62. 25904334 10.1091/mbc.E14-07-1194 PMC4462943

[47] Voetmann LM, Rolin B, Kirk RK, Pyke C, Hansen AK. The intestinal permeability marker FITC-dextran 4kDa should be dosed according to lean body mass in obese mice. Nutr Diabetes. 2023;13:1. 10.1038/s41387-022-00230-2 PMC9816099 36604407

[48] Gillis CC, Hughes ER, Spiga L, Winter MG, Zhu W, de Carvalho TF, et al. Dysbiosis-associated change in host metabolism generates lactate to support *Salmonella* growth. Cell Host Microbe. 2018;23(4):570. 10.1016/j.chom.2018.03.013 PMC5907491 29649446

[49] Taylor SJ, Winter MG, Gillis CC, Silva LAD, Dobbins AL, Muramatsu MK, et al. Colonocyte-derived lactate promotes E. coli fitness in the context of inflammation-associated gut microbiota dysbiosis. Microbiome. 2022;10:200. 10.1186/s40168-022-01389-7 PMC9701030 36434690

[50] Nabil NM, Tawakol MM, Samir A, Hassan HM, Yonis AE, Reda RM, et al. Synergistic influence of probiotic and florfenicol on embryonic viability, performance, and multidrug-resistant *Salmonella* Enteritidis in broiler chickens. Sci Rep. 2023;13:9644. 10.1038/s41598-023-36238-6 PMC10267169 37316527

[51] Zheng DP, Liwinski T, Elinav E. Interaction between microbiota and immunity in health and disease. Cell Res. 2020;30(6):492–506. 32433595 10.1038/s41422-020-0332-7 PMC7264227

[52] Hou K, Wu ZX, Chen XY, Wang JQ, Zhang D, Xiao C, et al. Microbiota in health and diseases. Signal Transduct Target Ther. 2022;7(1):135. 35461318 10.1038/s41392-022-00974-4 PMC9034083

[53] Ullah H, Arbab S, Tian YL, Chen YW, Liu CQ, Li QJ, et al. Crosstalk between gut microbiota and host immune system and its response to traumatic injury. Front Immunol. 2024;15:1413485. 39144142 10.3389/fimmu.2024.1413485 PMC11321976

[54] Das O, Masid A, Chakraborty M, Gope A, Dutta S, Bhaumik M. Butyrate driven raft disruption trots off enteric pathogen invasion: possible mechanism of colonization resistance. Gut Pathog. 2023;15(1):19. 37085870 10.1186/s13099-023-00545-0 PMC10122309

[55] Zhang T, Ding H, Chen L, Lin Y, Gong Y, Pan Z, et al. Antibiotic-induced dysbiosis of microbiota promotes chicken lipogenesis by altering metabolomics in the cecum. Metabolites. 2021;11(8):487. 34436428 10.3390/metabo11080487 PMC8398106

[56] Drummond RA, Desai JV, Ricotta EE, Swamydas M, Deming C, Conlan S, et al. Long-term antibiotic exposure promotes mortality after systemic fungal infection by driving lymphocyte dysfunction and systemic escape of commensal bacteria. Cell Host Microbe. 2022;30(7):1020–33 e6. 35568028 10.1016/j.chom.2022.04.013 PMC9283303

[57] Mei XR, Ma BH, Zhai XW, Zhang AY, Lei CW, Zuo L, et al. Florfenicol enhances colonization of a *Salmonella enterica* serovar Enteritidis *floR* mutant with major alterations to the intestinal microbiota and metabolome in neonatal chickens. Appl Environ Microb. 2021;87(24):e01681–e1721. 10.1128/AEM.01681-21 PMC8612288 34613752

[58] Rivera-Chávez F, Lopez CA, Bäumler AJ. Oxygen as a driver of gut dysbiosis. Free Radical Bio Med. 2017;105:93–101. 27677568 10.1016/j.freeradbiomed.2016.09.022

[59] Rath E, Haller D. Intestinal epithelial cell metabolism at the interface of microbial dysbiosis and tissue injury. Mucosal Immunol. 2022;15(4):595–604. 35534699 10.1038/s41385-022-00514-x PMC9259489

[60] Alber A, Morris KM, Bryson KJ, Sutton KM, Monson MS, Chintoan-Uta C, et al. Avian pathogenic *Escherichia coli* (APEC) strain-dependent immunomodulation of respiratory granulocytes and mononuclear phagocytes in CSF1R-reporter transgenic chickens. Front Immunol. 2019;10:3055. 31998322 10.3389/fimmu.2019.03055 PMC6967599

[61] Mol N, Peng L, Esnault E, Quere P, Haagsman HP, Veldhuizen EJA. Avian pathogenic *Escherichia coli* infection of a chicken lung epithelial cell line. Vet Immunol Immunopathol. 2019;210:55–9. 30947980 10.1016/j.vetimm.2019.03.007

[62] Ewers C, Antao EM, Diehl I, Philipp HC, Wieler LH. Intestine and environment of the chicken as reservoirs for extraintestinal pathogenic strains with zoonotic potential. Appl Environ Microb. 2009;75(1):184–92. 10.1128/AEM.01324-08 PMC2612213 18997030

[63] Mebius RE, Kraal G. Structure and function of the spleen. Nat Rev Immunol. 2005;5(8):606–16. 16056254 10.1038/nri1669

[64] Lewis SM, Williams A, Eisenbarth SC. Structure and function of the immune system in the spleen. Sci Immunol. 2019;4(33):eaau6085. 30824527 10.1126/sciimmunol.aau6085 PMC6495537

[65] Sutton KM, Morris KM, Borowska D, Sang H, Kaiser P, Balic A, et al. Characterization of conventional dendritic cells and macrophages in the spleen using the -reporter transgenic chickens. Front Immunol. 2021;12:636436.

[66] Meijerink N, van den Biggelaar R, van Haarlem DA, Stegeman JA, Rutten V, Jansen CA. A detailed analysis of innate and adaptive immune responsiveness upon infection with *Salmonella enterica* serotype Enteritidis in young broiler chickens. Vet Res. 2021;52:109. 10.1186/s13567-021-00978-y PMC8369617 34404469

[67] Shi J, Jiang S, Wang Q, Dong J, Zhu H, Wang P, et al. Spleen-based proteogenomics reveals that *Escherichia coli* infection induces activation of phagosome maturation pathway in chicken. Virulence. 2023;14(1):2150453. 36411420 10.1080/21505594.2022.2150453 PMC9817119

[68] Matsuyama-Kato A, Iseki H, Boodhoo N, Bavananthasivam J, Alqazlan N, Abdul-Careem MF, et al. Phenotypic characterization of gamma delta (γδ) T cells in chickens infected with or vaccinated against Marek’s disease virus. Virology. 2022;568:115–25. 10.1016/j.virol.2022.01.012 35152043

[69] Hartle S, Sutton K, Vervelde L, Dalgaard TS. Delineation of chicken immune markers in the era of omics and multicolor flow cytometry. Front Vet Sci. 2024;11:1385400. 38846783 10.3389/fvets.2024.1385400 PMC11156169

[70] Papotto PH, Yilmaz B, Pimenta G, Mensurado S, Cunha C, Fiala GJ, et al. Maternal γδ T cells shape offspring pulmonary type 2 immunity in a microbiota-dependent manner. Cell Rep. 2023;42(2):112074. 10.1016/j.celrep.2023.112074 PMC7615642 36787741

[71] Dupraz L, Magniez A, Rolhion N, Richard ML, Da Costa G, Touch S, et al. Gut microbiota-derived short-chain fatty acids regulate IL-17 production by mouse and human intestinal gammadelta T cells. Cell Rep. 2021;36(1):109332. 34233192 10.1016/j.celrep.2021.109332

[72] Luu M, Riester Z, Baldrich A, Reichardt N, Yuille S, Busetti A, et al. Microbial short-chain fatty acids modulate CD8^+^ T cell responses and improve adoptive immunotherapy for cancer. Nat Commun. 2021;12:4077. 10.1038/s41467-021-24331-1 PMC8249424 34210970

[73] Duan H, Wang L, Huangfu M, Li H. The impact of microbiota-derived short-chain fatty acids on macrophage activities in disease: Mechanisms and therapeutic potentials. Biomed Pharmacother. 2023;165:115276. 37542852 10.1016/j.biopha.2023.115276

[74] Lee IK, Gu MJ, Ko KH, Bae S, Kim G, Jin GD, et al. Regulation of CD4^+^CD8^−^CD25^+^ and CD4^+^CD8^+^CD25^+^ T cells by gut microbiota in chicken. Sci Rep. 2018;8:8627. 10.1038/s41598-018-26763-0 PMC5988814 29872084

[75] Selvaraj RK. Avian CD4^+^CD25^+^ regulatory T cells: properties and therapeutic applications. Dev Comp Immunol. 2013;41(3):397–402. 10.1016/j.dci.2013.04.018 23665004

[76] Lv X, Hao J, Wu L, Liu M, He L, Qiao Y, et al. Age quadratically affects intestinal calcium and phosphorus transporter gene expression in broiler chickens. Anim Biosci. 2022;35(12):1921–8. 35507840 10.5713/ab.22.0058 PMC9659444

[77] Cox LM, Yamanishi S, Sohn J, Alekseyenko AV, Leung JM, Cho I, et al. Altering the intestinal microbiota during a critical developmental window has lasting metabolic consequences. Cell. 2014;158(4):705–21. 25126780 10.1016/j.cell.2014.05.052 PMC4134513

[78] Ignacio A, Czyz S, McCoy KD. Early life microbiome influences on development of the mucosal innate immune system. Semin Immunol. 2024;73:101885. 38788491 10.1016/j.smim.2024.101885

[79] Choi S, Kim EB. A comprehensive longitudinal study of gut microbiota dynamic changes in laying hens at four growth stages prior to egg production. Anim Biosci. 2023;36(11):1727–37. 37871901 10.5713/ab.23.0271 PMC10623045

[80] Lynn MA, Eden G, Ryan FJ, Bensalem J, Wang X, Blake SJ, et al. The composition of the gut microbiota following early-life antibiotic exposure affects host health and longevity in later life. Cell Rep. 2021;36(8):109564. 34433065 10.1016/j.celrep.2021.109564

[81] Perdijk O, Butler A, Macowan M, Chatzis R, Bulanda E, Grant RD, et al. Antibiotic-driven dysbiosis in early life disrupts indole-3-propionic acid production and exacerbates allergic airway inflammation in adulthood. Immunity. 2024;57(8):1939–54 e7. 39013465 10.1016/j.immuni.2024.06.010

[82] Miao ZH, Zhou WX, Cheng RY, Liang HJ, Jiang FL, Shen X, et al. Dysbiosis of intestinal microbiota in early life aggravates high-fat diet induced dysmetabolism in adult mice. BMC Microbiol. 2021;21:209. 10.1186/s12866-021-02263-6 PMC8268513 34238228

[83] Fu Y, Hu J, Erasmus MA, Johnson TA, Cheng HW. Effects of early-life cecal microbiota transplantation from divergently selected inbred chicken lines on growth, gut serotonin, and immune parameters in recipient chickens. Poult Sci. 2022;101(7):101925. 35613492 10.1016/j.psj.2022.101925 PMC9130533

[84] Tabilas C, Iu DS, Daly CWP, Yee Mon KJ, Reynaldi A, Wesnak SP, et al. Early microbial exposure shapes adult immunity by altering CD8 T cell development. Proc Natl Acad Sci U S A. 2022;119(49):e2212548119. 10.1073/pnas.2212548119 PMC9894172 36442114

[85] Di Chiara C, Boracchini R, Cantarutti A, Kakkar F, Oletto A, Padoan A, et al. Risk of SARS-CoV-2 reinfection in children within the 12 months following mild COVID-19: Insights from a survey study. Pediatr Infect Dis J. 2024;43(4):e128–30. 38241645 10.1097/INF.0000000000004233 PMC10919262

