# Supplementary figures and images for: Florfenicol-induced dysbiosis impairs intestinal homeostasis and host immune system in laying hens
Source: J Anim Sci Biotechnol. 2025 Apr 14;16:56. doi: 10.1186/s40104-025-01186-w (PMC11995664; doi:10.1186/s40104-025-01186-w)

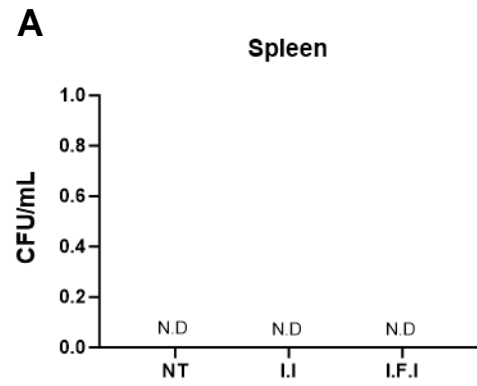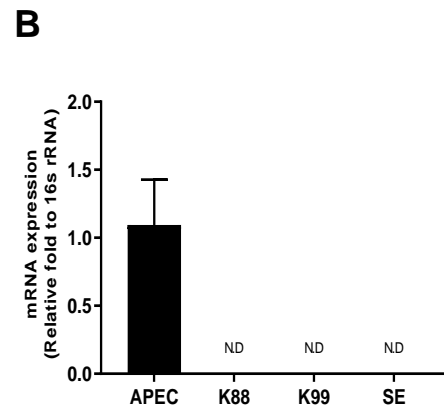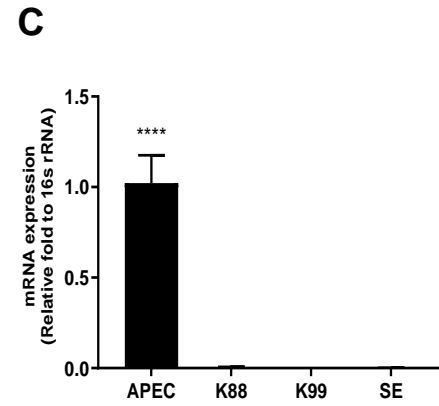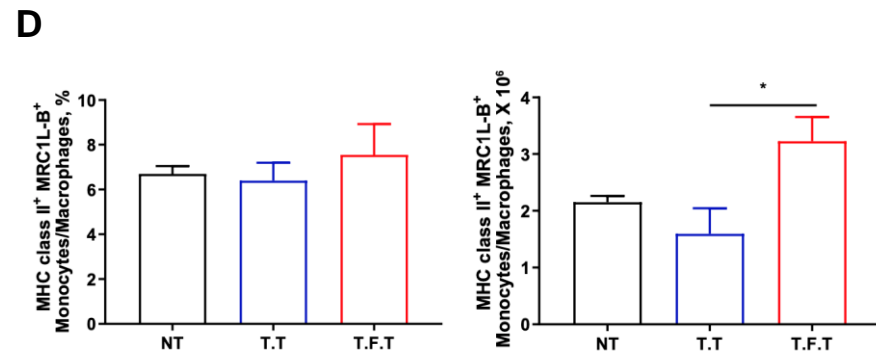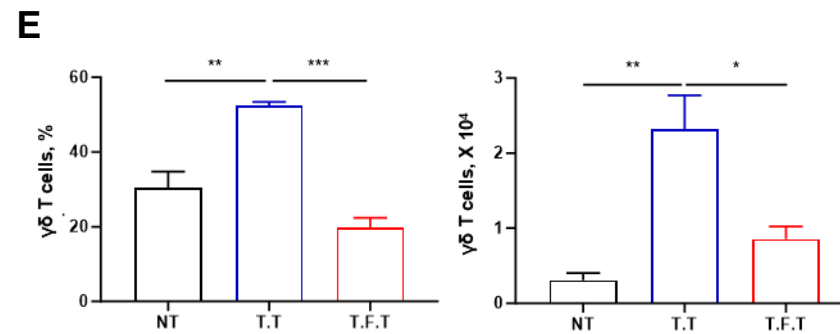

Supplement: Supplementary file 1 — Additional file1: Fig. S1. Florfenicol-induced dysbiosis increases susceptibility to systemic APEC infection. Chickens (n = 6) were infected with APEC 3 d prior to florfenicol treatment. Following the withdrawal phase, they were re-infected with APEC. Systemic infection at 1 dpi was quantified by determining (A) the mean log10 CFU/mL in the spleen and the levels of (B) wzx and (C) neuC1 mRNA in bacteria isolated from APEC O1:K1, E. coli K88 and K99, and Salmonella Enteritidis. Changes in the percentage and absolute number of (D) splenic macrophages and (E) lamina propria γδ T cells. NT, non-treated. T.T, APEC double infection without florfenicol treatment. T.F.T, APEC double infection with florfenicol treatment. Statistical differences were determined in a Tukey test; *P < 0.05, **P < 0.01, ***P < 0.001. [file 40104_2025_1186_MOESM1_ESM.pdf]

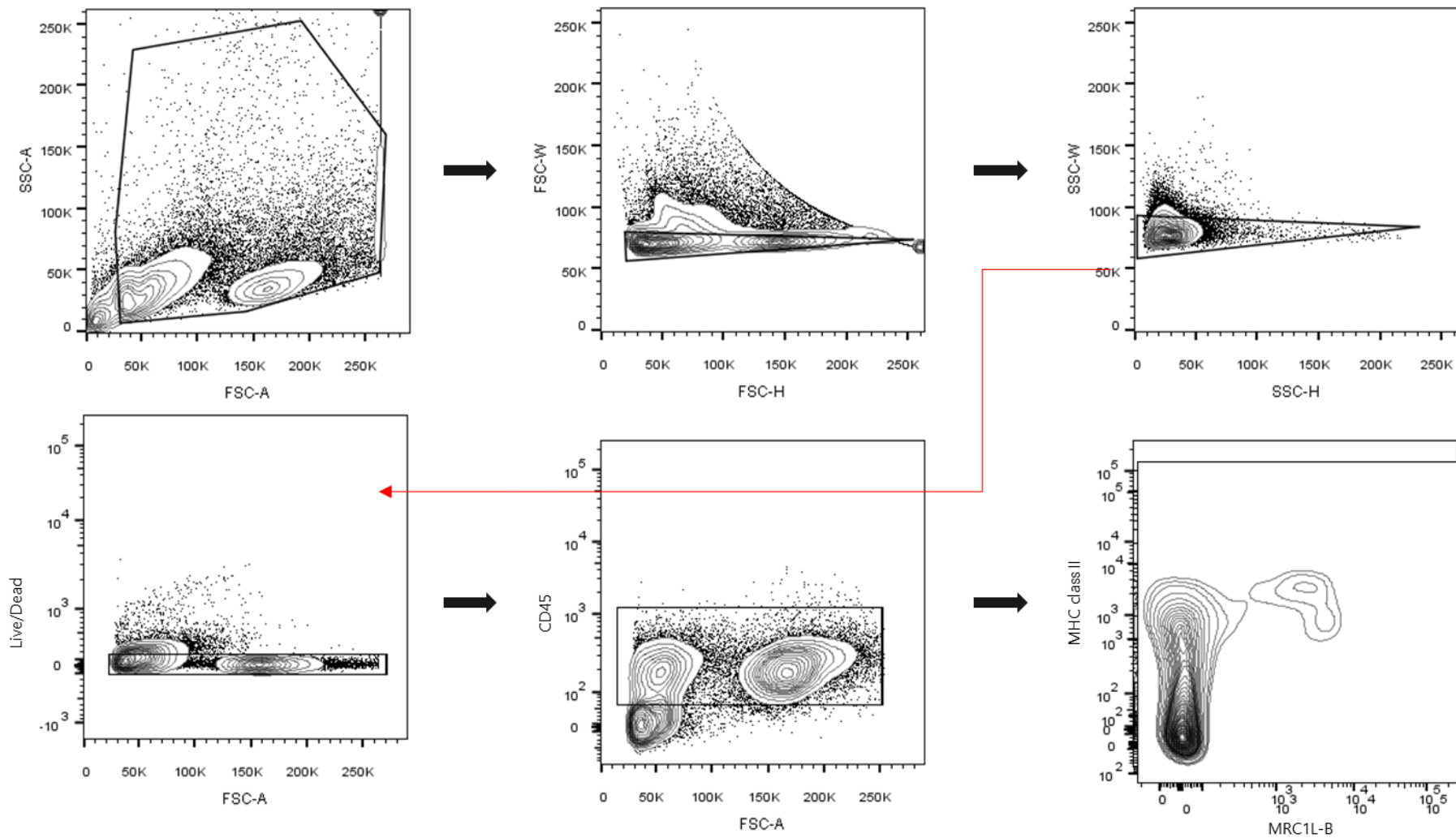

Supplement: Supplementary file 2 — Additional file 2: Fig. S2. Gating strategy for splenic monocytes/macrophages. Live single splenocytes were gated by FSC-A vs. SSC-A based on CD45 expression, followed by the gating of monocytes/macrophages based on MHC class II and MRC1L-B expression. [file 40104_2025_1186_MOESM2_ESM.pdf]

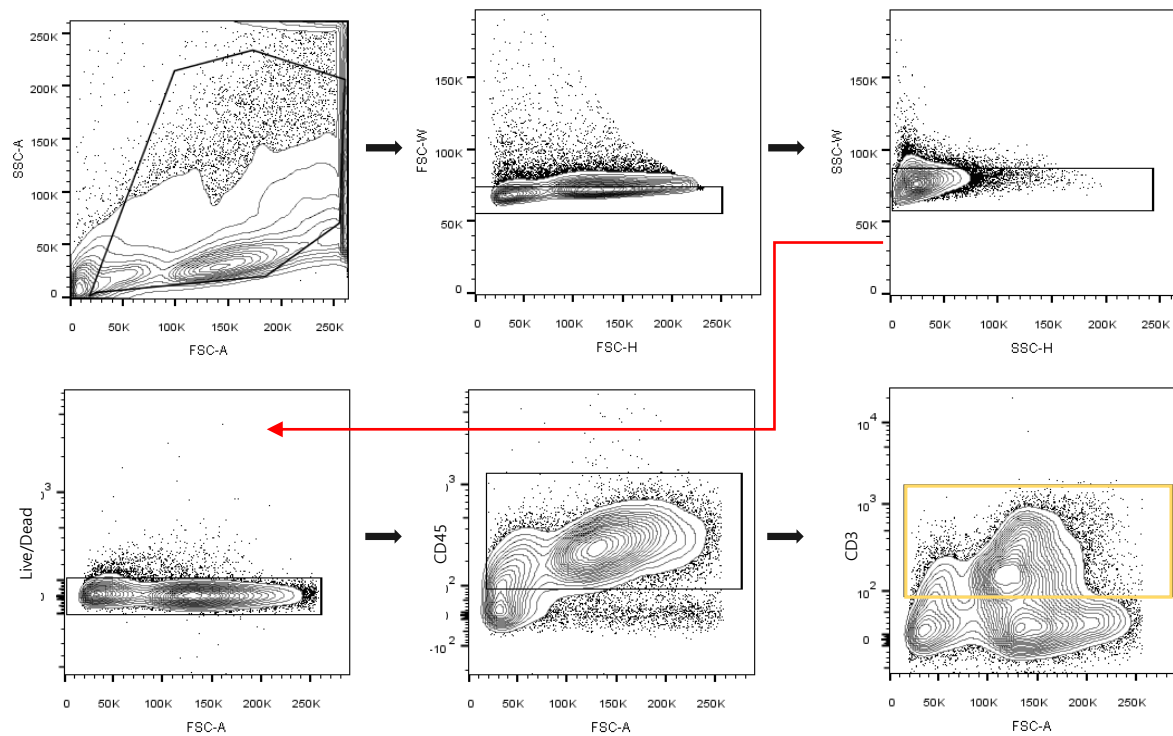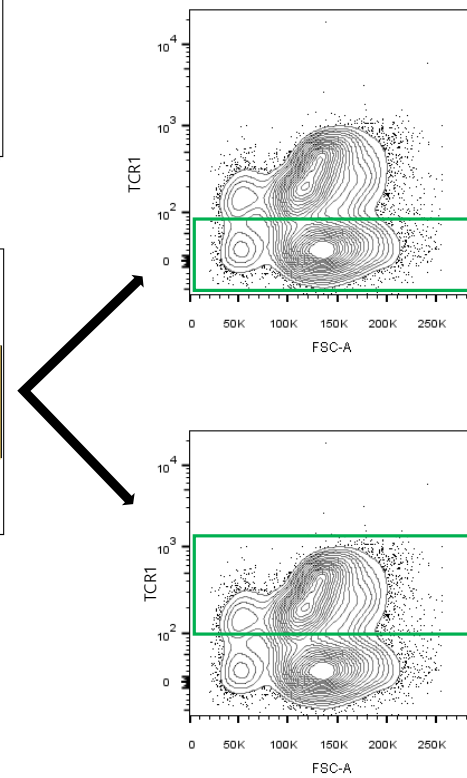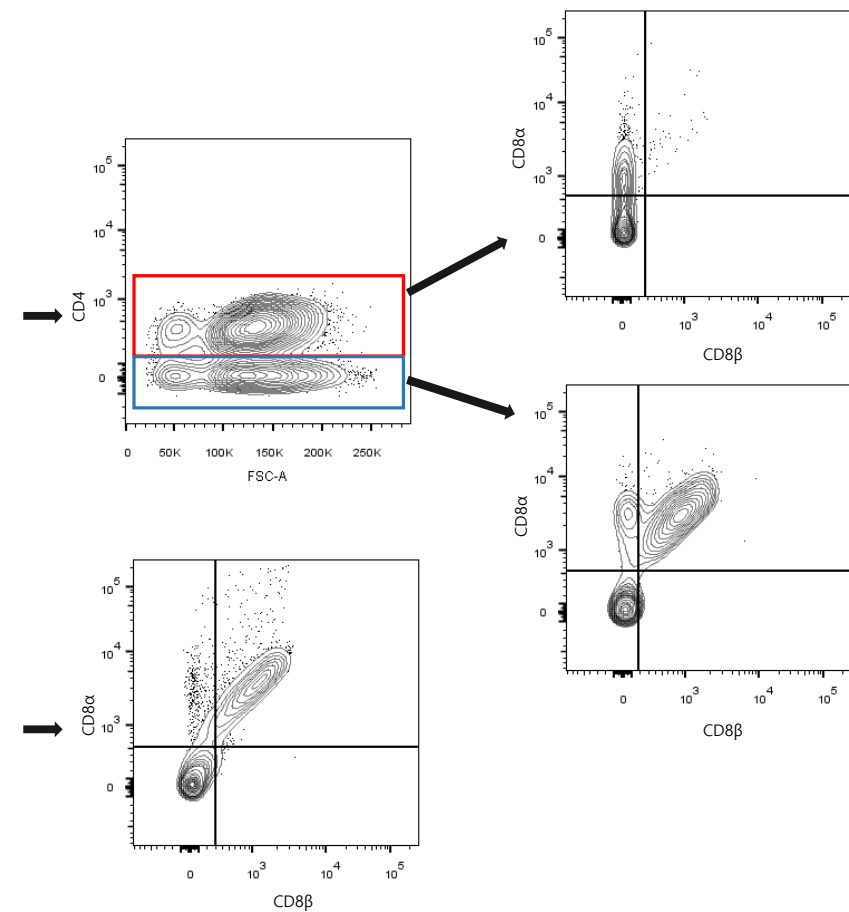

Supplement: Supplementary file 3 — Additional file 3: Fig. S3. Gating strategy T cells in the lamina propria. Live single cells were gated by FSC-A vs. SSC-A. Single cells from lamina propria were gated based on CD45 expression , followed by CD3 to identify T cells. T cells were divided into γδ T cells and non-γδ T cells based on TCR γδ (TCR1) expression, with γδ T cells then sub-divided into three subpopulations. Non-γδ T cells were sub-divided based on CD4 expression. CD4+ and CD4– T cells were then sub-divided into subpopulations based on CD8a or CD8b expression. [file 40104_2025_1186_MOESM3_ESM.pdf]

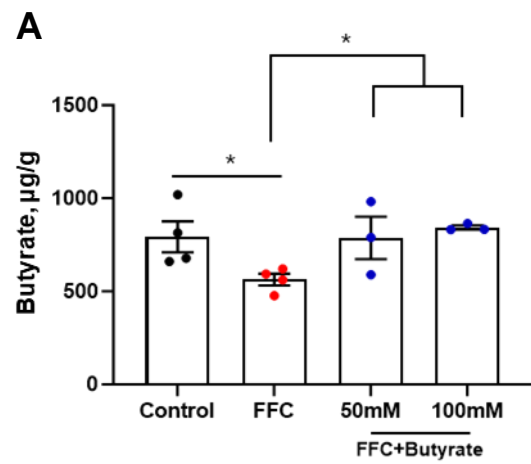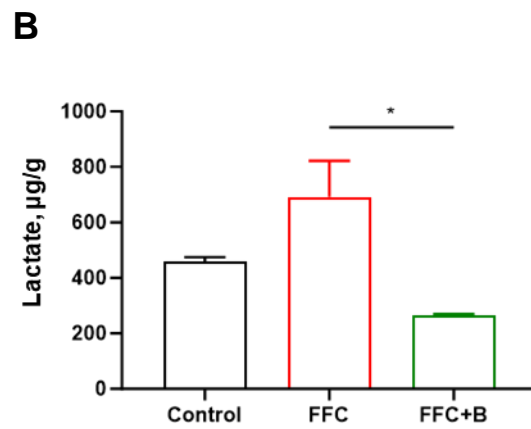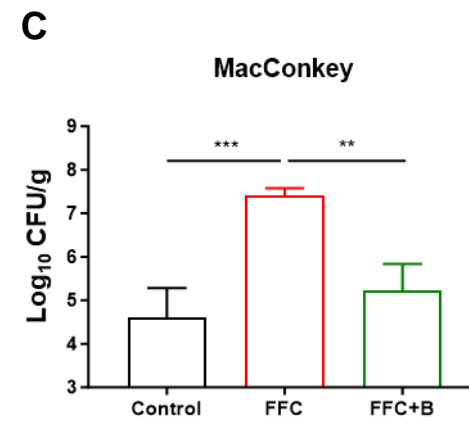

Supplement: Supplementary file 4 — Additional file 4: Fig. S4. Butyrate administration restores gut homeostasis impaired by florfenicol treatment. To achieve butyrate levels similar to those under homeostasis conditions in the presence of florfenicol-induced dysbiosis, chickens were provided with 50 mmol/L or 100 mmol/L butyrate in their drinking water. (A) Butyrate levels in the cecum were then measured. (B) Alteration of cecal lactate levels measured by HPLC. (C) Pathobionts in the cecal content from control, florfenicol, and florfenicol+butyrate groups as measured based on CFU counts on MacConkey agar. FFC, florfenicol. FFC+B, florfenicol+butyrate. Statistical differences were determined in a Tukey test; *P < 0.05, **P < 0.01, ***P < 0.001. [file 40104_2025_1186_MOESM4_ESM.pdf]

**A**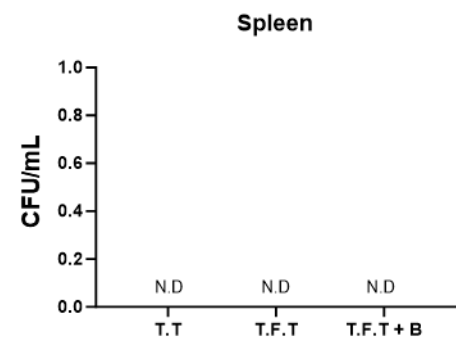**B**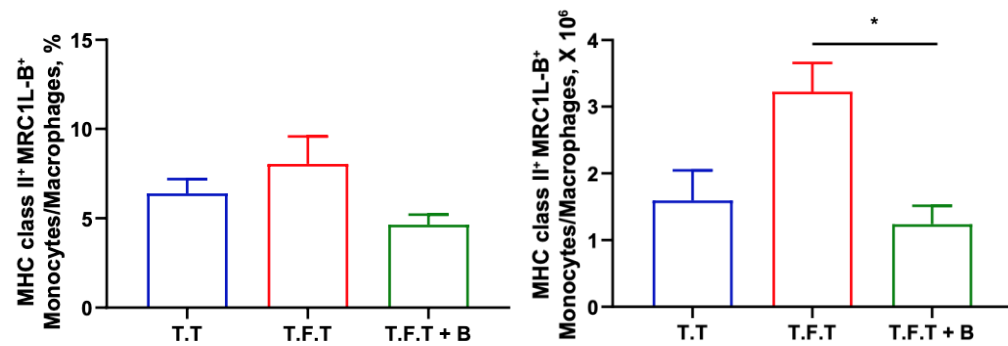**C**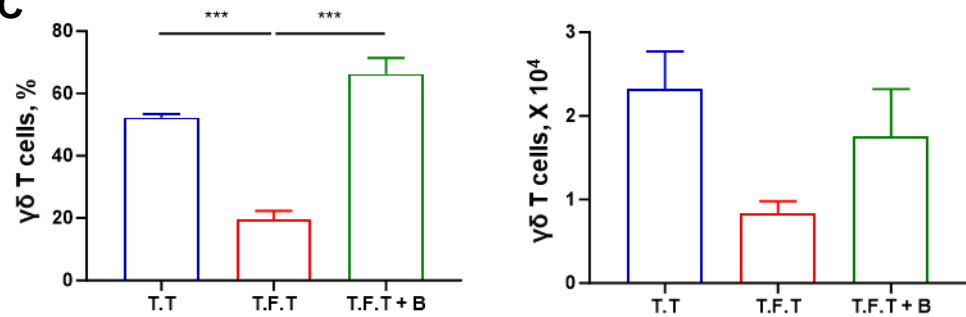

Supplement: Supplementary file 5 — Additional file 5: Fig. S5. Butyrate administration restores the susceptibility to systemic APEC infection. Butyrate was administered to chickens in their drinking water starting with the first APEC infection until 3 d after re-infection. Systemic infection of APEC was quantified at 1 dpi by determining the (A) mean log10 CFU/mL in the spleen and the changes in the percentage and absolute number of (B) splenic macrophages and (C) lamina propria γδ T cells. T.T, APEC double infection without florfenicol treatment. T.F.T, APEC double infection with florfenicol treatment. T.F.T+B APEC double infection with florfenicol and butyrate treatment. Statistical differences were determined in a Tukey test; *P < 0.05, ***P < 0.001. [file 40104_2025_1186_MOESM5_ESM.pdf]

***Claudin-1***

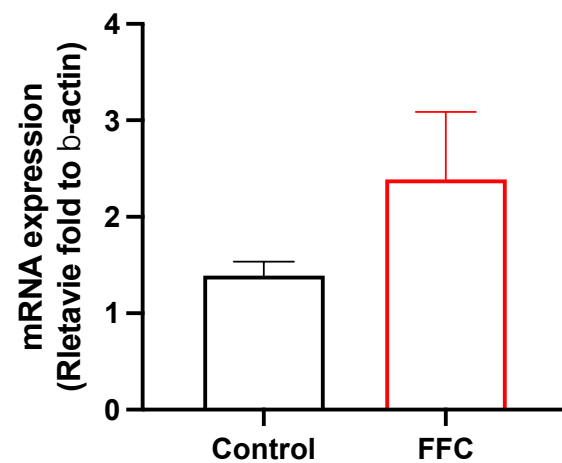

***Claudin-2***

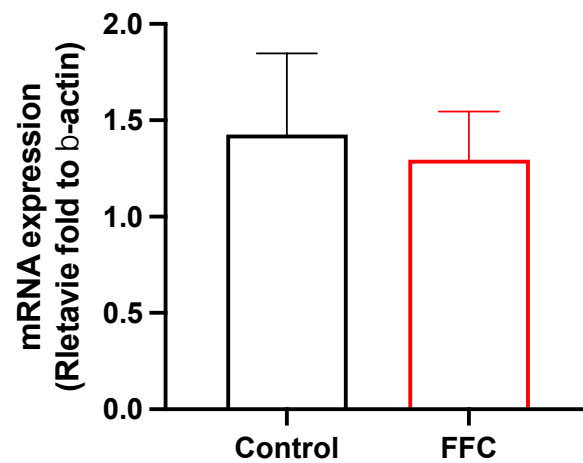

***Claudin-3***

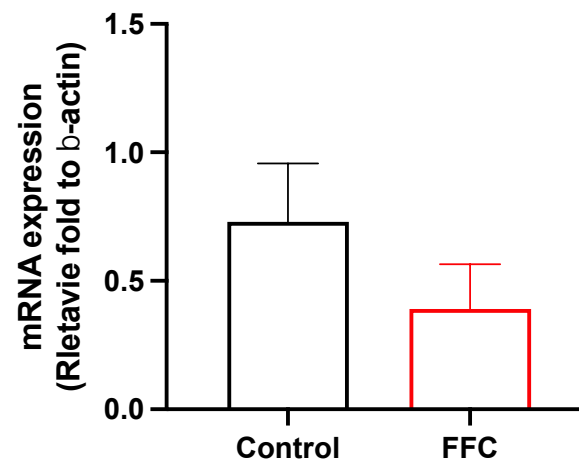

***ZO-1***

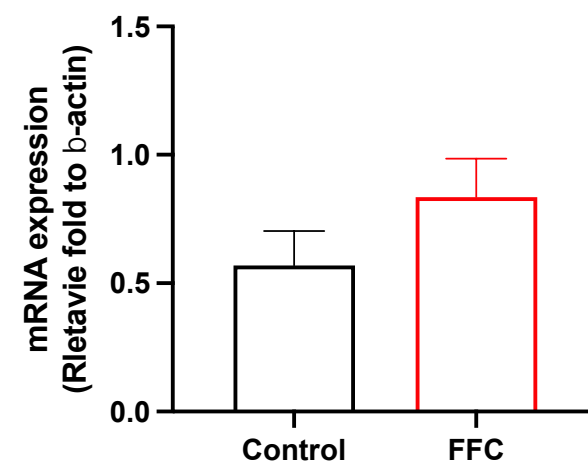

Supplement: Supplementary file 6 — Additional file 6: Fig. S6. Claudin-1, -2, -3, and ZO-1 are not affected by florfenicol treatment. The mRNA expression levels of Claudin-1, -2, -3, and ZO-1 in chickens treated with PBS or florfenicol. FFC, florfenicol. Results are presented as the mean ± SEM. [file 40104_2025_1186_MOESM6_ESM.pdf]
